# An Amazing 30-Year Journey around the DABO Family: A Medicinal Chemistry Lesson on a Versatile Class of Non-nucleoside HIV-1 Reverse Transcriptase Inhibitors

**DOI:** 10.1021/acs.jmedchem.4c02848

**Published:** 2025-03-07

**Authors:** Emanuele Fabbrizi, Vladimir V. Chernyshov, Francesco Fiorentino, Gianluca Sbardella, Rino Ragno, Maxim Nawrozkij, Roman Ivanov, Dante Rotili, Antonello Mai

**Affiliations:** † Department of Drug Chemistry and Technologies, Sapienza University of Rome, Piazzale Aldo Moro 5, 00185 Rome, Italy; # Sirius University of Science and Technology, Olympic Avenue, 1, 354340, Federal Territory of Sirius, Krasnodar Region Russian Federation; + Department of Pharmacy, University of Salerno, via Giovanni Paolo II 132, 84084 Fisciano, SA, Italy; § Department of Science, Roma Tre University of Rome, Viale Guglielmo Marconi 446, 00146 Rome, Italy

## Abstract

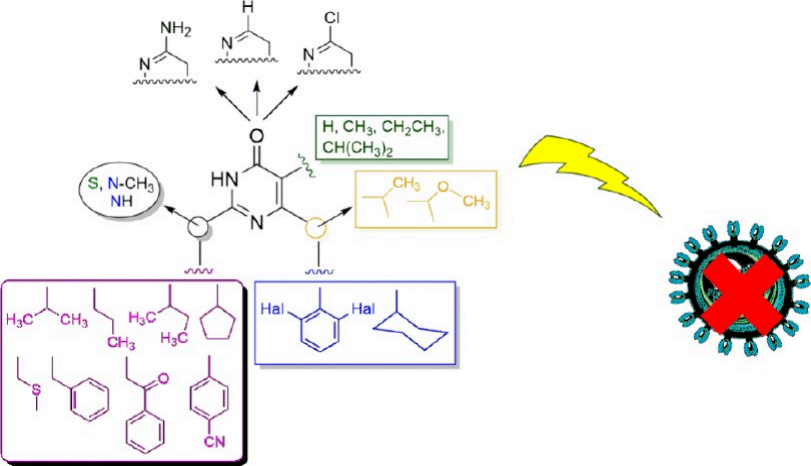

Since the emergence of AIDS, the non-nucleoside HIV-1 RT inhibitors (NNRTIs) have attracted the attention of scientists and clinicians due to their high potency and specificity combined with low toxicity. 3,4-Dihydro-2-alkoxy-6-benzyl-4-oxopyrimidines (DABOs) are a family of NNRTIs described since 1992, and the best members among *S*-, *NH-*, and *N*,*N*-DABOs showed high anti-HIV-1 potency in both cellular and enzymatic assays. During 30 years of research, the central 4-(3*H*)-pyrimidinone nucleus has been decorated with 2,6-dihaloaryl or cyclohexyl groups at the methylene at C6, alkyl- or (arylalkyl/aroylalkyl)thio/amino chains at C2, and hydrogen or a small alkyl group at C5. The further introduction of small (i.e., methoxy) groups at the C6 α-benzylic position furnished potency at the sub-nanomolar level against wild-type HIV-1 and at the nanomolar level against HIV-1 mutant strains. Importantly, some compounds of the DABO family exhibited preventative microbicidal activity, valuable in clinical settings where oral adherence rates are low.

## Significance

- HIV non-nucleoside reverse transcriptase inhibitors display high potency and specificity combined with low toxicity playing a crucial role in highly active antiretroviral therapies
- 3,4-Dihydro-2-alkoxy-6-benzyl-4-oxopyrimidines (DABOs) are a family of HIV-1 non-nucleoside reverse transcriptase inhibitors (NNRTIs) described since 1992
- Many authors around the world determined structure–activity relationships optimizing their potency up to sub-nanomolar level
- Long-acting NNRTI formulations are promising for HIV prevention, providing controlled release at transmission sites and improving adherence compared to daily oral doses

## 1 Introduction

Speaking about an epistemic view of medicinal chemistry, in 2015 Baier and Stahl wondered how many compounds it takes to tell a story.^1^ Although it is difficult to give an unambiguous answer to this question, looking back on the anthology of the so-called DABO (3,4-dihydro-2-alkoxy-6-benzyl-4-oxopyrimidine) family of HIV-1 non-nucleoside reverse transcriptase inhibitors (NNRTIs),^2^ it seems clear that it deserves its own story.

### 1.1 The HIV-1 Reverse Transcriptase (RT)

**Figure 1:**
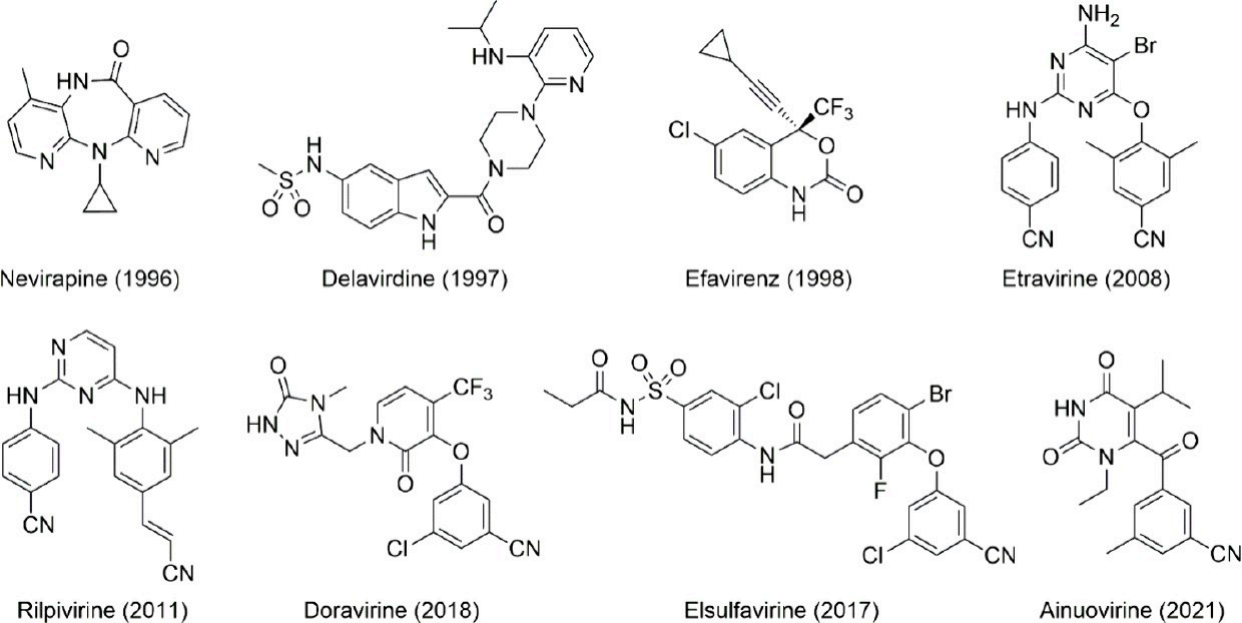
Six NNRTIs approved by the FDA (nevirapine, delavirdine, efavirenz, etravirine, rilpivirine, and doravirine) and those approved in Russia (elsulfavirine) and in China (ainuovirine) for the treatment of HIV infection.

The HIV-1 RT is a multifunctional enzyme responsible for the conversion of viral single-stranded RNA into double-stranded DNA, a process essential for the viral replication cycle. Due to the absence of analogous proteins in humans, RT represents an ideal target for developing anti-HIV-1 drugs, and RT inhibitors are a cornerstone of Highly Active Antiretroviral Therapy (HAART) to fight AIDS. Currently, two classes of RT inhibitors are utilized in clinical practice: nucleoside RT inhibitors (NRTIs) and NNRTIs. However, the therapeutic use of the former is hampered by issues such as toxicity, poor pharmacokinetics, and the emergence of resistant viral strains, while NNRTIs are characterized by high antiviral potency and negligible cytotoxicity, thus representing a valuable weapon against HIV-1 infection. To date, six NNRTIs have been approved by the Food and Drug Administration, the “first generation” drugs nevirapine, delavirdine, and efavirenz, and the “second generation” etravirine, rilpivirine, and doravirine (Figure 1). Two further NNRTIs, elsulfavirine and ainuovirine, have been approved by the regulatory Russian and Chinese organizations, respectively (Figure 1).^3,4^ They are widely utilized in clinical practice and contribute significantly to improving the quality of life of HIV patients.^5^ However, the swift emergence of drug resistance has constrained their effectiveness, highlighting the ongoing need for research and the development of innovative therapeutic options.

### 1.2 The Beginning of the Story

**Figure 2:**
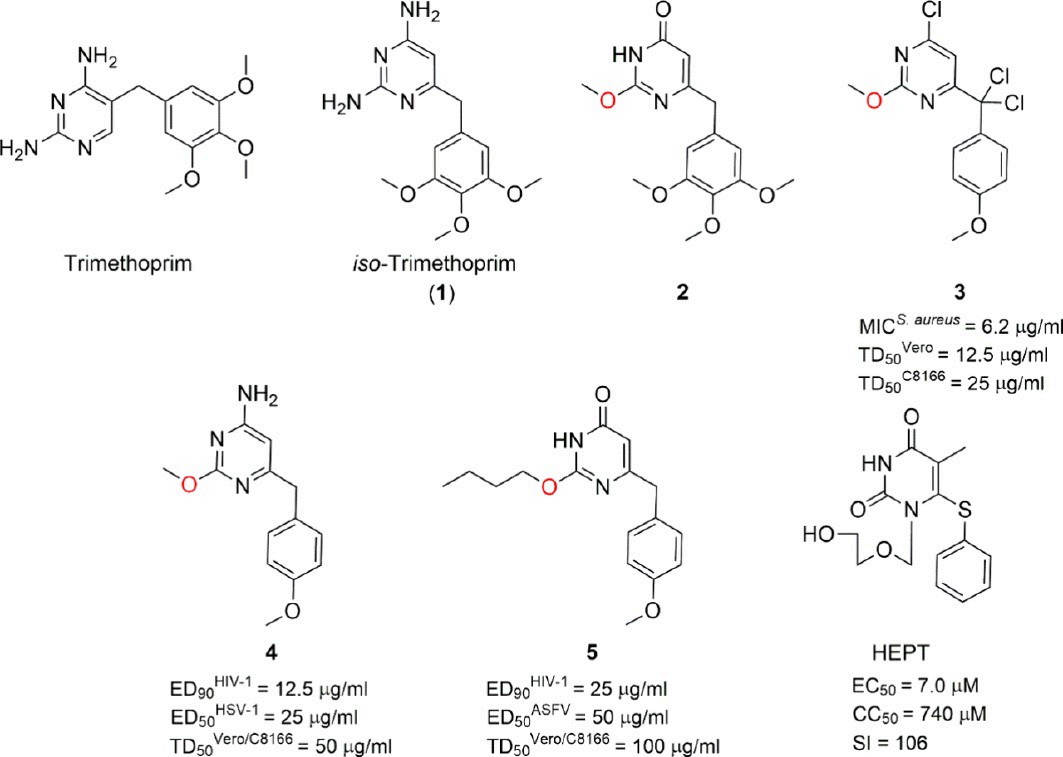
First 6-benzylpyrimidines analogues of trimethoprim tested as antimicrobial and antiviral agents. Chemical structure, anti-HIV-1 activity (EC_50_, effective concentration of compound required to achieve 50% protection of cells against the cytopathic effect of HIV-1), cytotoxicity (CC_50_, cytotoxic concentration of compound required to reduce the viability of mock-infected cells by 50%), and selectivity index (SI, ratio of CC_50_/EC_50_) of HEPT are also shown.

In the mid-1980s, Botta, Artico et al.^6^ described the synthesis of iso-trimethoprim **1**, a regioisomer of the well-known antimalarial drug trimethoprim differing by the shift of the 3,4,5-trimethoxybenzyl substituent from the C5 to the C6 position of the pyrimidine nucleus. In this process, the cyclocondensation of *in situ* generated *O-*methylisourea with ethyl 3-oxo-4-(3,4,5-trimethoxylphenyl)butanoate led to the 2-methoxy-6-(3,4,5-trimethoxybenzyl)pyrimidin-4(3*H*)-one **2**, which was subsequently converted into **1** via treatment with Vilsmeier–Haack reagent followed by ammonolysis of the corresponding 4-chloropyrimidine derivative. Further chemical manipulation of **2** generated various analogs of **1**, such as compounds **3**–**5**, which were tested as antimicrobial and antiviral agents using trimethoprim, methotrexate, and sulfamethoxazole as reference drugs (Figure 2).^6^

Despite their structural similarity to trimethoprim, the newly synthesized compounds showed negligible activity against the fungi and bacteria tested (*Candida albicans*, *Staphylococcus aureus*, *Escherichia coli*, and *Streptococcus mitis*), with the only exception of the 4-chloro-6-[dichloro(4-methoxyphenyl)methyl]-2-methoxypyrimidine **3** which was as potent as trimethoprim and 20-fold more potent than sulfamethoxazole against *S. aureus.* The antiviral screening [against HIV-1, HSV-1, African swine fever virus (ASFV), and polio type 1 (Sabin strain, Sb-1)] led to a bit more encouraging results. Two of the newly prepared compounds, **4** and **5**, showed little anti-HIV-1 activity, inhibiting its cytopathic effect (that is, structural changes in a host cell resulting from viral infection) by 90% at concentrations of 12.5 and 25 μg/mL, respectively. They were much less potent than AZT, used as a reference drug, but this modest activity joined to the structure similarity observed between these compounds and 1-[(2-hydroxyethoxy)methyl]-6-(phenylthio)thymine (HEPT)^7^ (Figure 2), a reliable anti-HIV-1 agent described immediately before, prompted the involved research teams to continue with this research line.

## 2 From DABOs to *S*-DABOs

Thus, in 1993–1995, a series of DABO derivatives were disclosed as a novel class of HIV-1 replication inhibitors in infected cells, acting by blocking the activity of the viral RT.^8−10^ Nucleophilic displacement of the C2-methoxy moiety of 2-methoxy-6-benzyl-4(3*H*)-pyrimidinone and its 5-methyl and 5-ethyl counterparts, all analogs of **2** and **5**, with a series of linear, branched, and cyclic alkoxy groups provided a series of 2-alkoxy-6-benzyl-4(3*H*)-pyrimidinones bearing a hydrogen, methyl, or ethyl group at the C5 position. Such compounds were screened against HIV-1 in infected MT-4 cells using HEPT, its 6-benzyl analog, 2′,3′-dideoxyinosine (ddI), and AZT as reference drugs.

**Figure 3:**
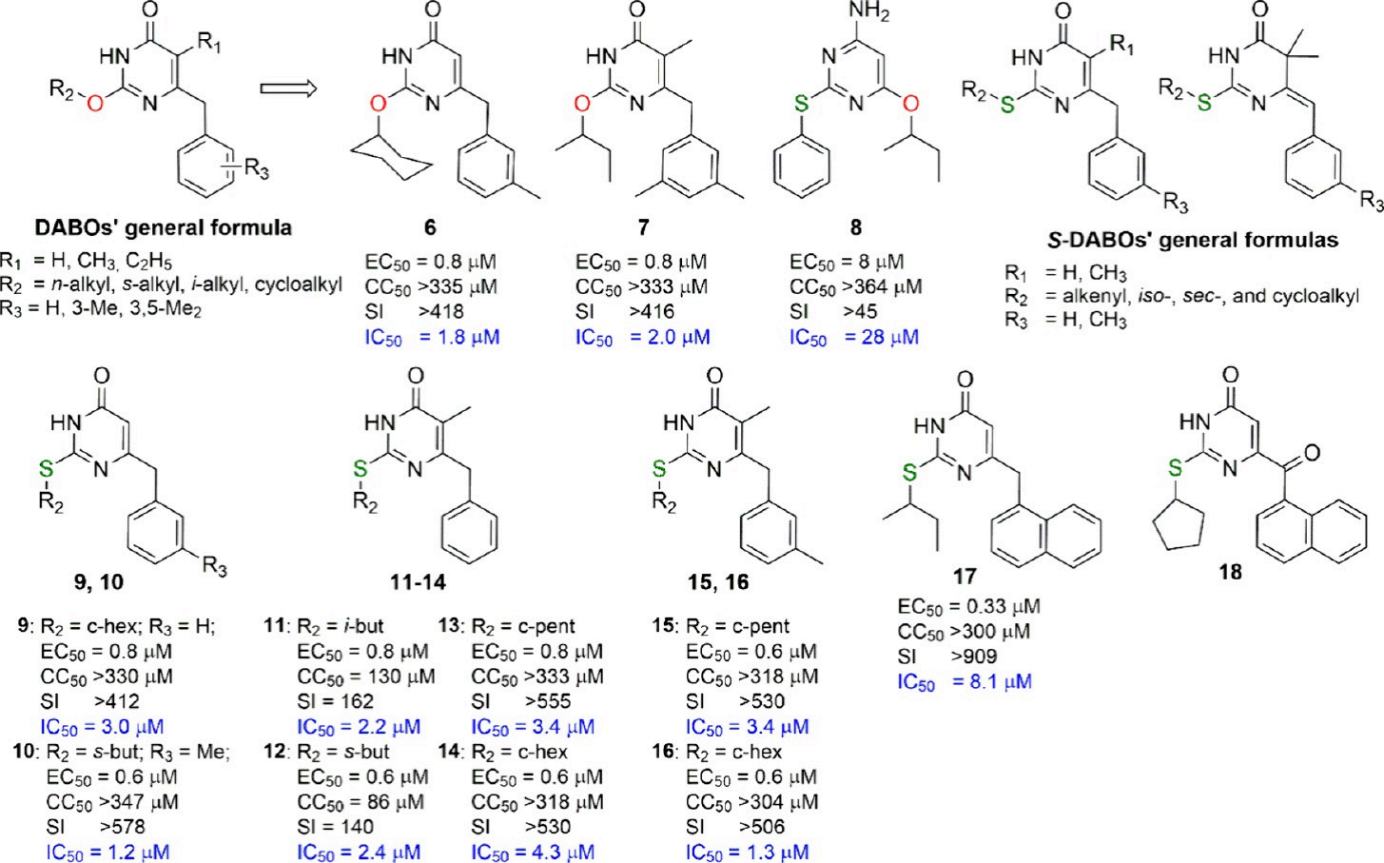
First examples of DABOs, *S*-DABOs, and DATNO derivatives. For each compound, the EC_50_ (effective concentration able to induce 50% inhibition of HIV-1 cytopathic effect in MT-4 cells), CC_50_ (cytotoxic concentration able to reduce by 50% the viability of mock-infected cells), SI (selectivity index, ratio of CC_50_/EC_50_), and IC_50_ (concentration of compound able to inhibit by 50% the HIV-1 RT activity, in blue) values are reported.

In general, these compounds displayed low cytotoxicity (CC_50_, cytotoxic concentration of compound required to reduce the viability of mock-infected MT-4 cells by 50%, from 77 to >1000 μM), and exhibited protection against HIV-1 (EC_50_, effective concentration of compound required to achieve 50% protection of MT-4 cells against the cytopathic effect of HIV-1) down to the sub-micromolar range, with selectivity indexes (SIs, ratios of CC_50_/EC_50_) >400 in the best cases (**6** and **7**; Figure 3). Compared with HEPT, **6** and **7** were approximately 9-fold more potent and 4-fold more selective as anti-HIV-1 agents. Against recombinant RT (rRT), the compounds showed single digit micromolar inhibition (IC_50_, concentration of compound able to inhibit the HIV-1 rRT activity by 50%), close to their cellular effective concentration. The antiviral activity of these compounds depended strongly on the nature of the *O*^2^-alkyl side chain, with those carrying *sec*- and iso-butyl, cyclopentyl, and cyclohexyl chains being the most potent. The 2-methoxy and *O*^2^-unsubstituted compounds were inactive or poorly potent. It is interesting to point out that the 2-*sec*-butyl substituent may be regarded as an open-chain equivalent of the cyclopentyl/cyclohexyl group. The introduction of one or two methyl groups at the 3 or 3,5 position of the C6-benzyl group increased the potency of the derivatives, indicating the importance of a substitution at the benzyl level. Since among the C5–H analogues the cyclohexyloxy substitution at C2 seemed to yield the most potent compounds whereas for the C5-methyl derivatives the *sec*-butyl group seemed to be preferred, a sort of balance of hydrophilicity/lipophilicity of these derivatives can be postulated to fit well the lipophilic RT binding site.

At the same time, HEPT/DABO hybrids were reported, in which the DABO benzyl portion was replaced by the isosteric phenoxy- or phenylthio-moiety and shifted from the C6 to the C2 pyrimidine position, whereas the alkoxy chain was moved from C2 to C6, according to a replacement later called the Sheridan criterion.^11,12^ Moreover, the C4-oxo group was converted to a primary amine substituent. Unfortunately, such a combination appeared to fail in terms of structure–activity relationships (SARs): the only compound active in the single digit micromolar range was the 4-amino-6-*sec*-butoxy-2-(phenylthio)pyrimidine **8** (Figure 3), while all of the other analogs appeared to be much less potent or completely inactive.

As a first follow-up to the DABOs, new analogs bearing the isosteric *O*-to-*S* replacement at the C2 position (*S*-DABOs) were reported in 1995 to further expand the SAR data (Figure 3).^13^*S*-DABOs were also designed and synthesized to make the compounds more synthetically accessible. Indeed, the alcoholysis of the 2-methoxy-4(3*H*)-pyrimidinones using a large excess of alkoxide, together with the need for chromatographic separation of the title compounds in the pure state, posed a problem for the DABOs’ further scalable production. Sulfur analogues proved to be the way forward for the further development of this class of compounds. Some reported *S*-DABOs showed inhibitory potency in the sub-micromolar range against HIV-1-infected MT-4 cells, in detail, compounds with (i) *sec*-butylthio, iso-butylthio, cyclopentylthio, and cyclohexylthio groups at C2, (ii) the benzyl or 3-methylbenzyl moiety at C6, and (iii) hydrogen or methyl substitution at C5 (see **9**–**16** in Figure 3). Cytotoxicity was very low for almost all compounds with high SIs.

In 1997, chemical manipulation applied at the N3 and C4 positions of the 2-(cyclohexylthio)-5-methyl-6-(3-methylbenzyl)-4(3*H*)-pyrimidinone **16** provided only inactive compounds with increased cytotoxicity, the most intriguing being the inactive 4-amino analog 2-(cyclohexylthio)-5-methyl-6-(3-methylbenzyl)-4-pyrimidinamine, which is a direct structural congener of the previously reported 2-methoxy-6-(4-methoxybenzyl)-4-pyrimidinamine **4** (Figure 2),^6^ from which the whole story began. Investigation on the C6 pyrimidine substituent through elongating or shortening the distance between the pyrimidine and benzene rings led to less potent derivatives, while replacement of benzyl with a 1- or, to a lesser extent, 2-naphthylmethyl moiety gave compounds [DATNOs, 3,4-dihydro-2-(alkylthio)-6-(naphthylmethyl)-4-oxopyrimidines] as potent as their 3-methylbenzyl counterparts or, in the case of the 2-(*sec*-butylthio)-6-(naphthalen-1-ylmethyl)-4(3*H*)-pyrimidinone **17**, slightly more potent.^14^ This structural modification of *S*-DABOs has a historical parallel with the work of Libermann and Hengl,^15^ dedicated to the structural optimization of the antithyroid 6-benzyl-2-thiouracils by substitution of the benzyl with the bioisosteric naphthylmethyl groups.

During this period, the X-ray crystal structures of two *S*-DABOs and one DATNO were reported, confirming the butterfly like orientation of the two aromatic and heterocyclic rings, typical of many other first-generation NNRTIs.^16,17^ It is worth mentioning that these products crystallize with difficulty, returning crystals of poor quality, and we had to wait until 2006 to have a new crystal structure, the *S*-cyclopentylthio-6-(1-naphthoyl)-4(3*H*)-pyrimidinone **18** (Figure 3), reported by Chen.^18^ This circumstance outlines one of the main Achilles’ heels of *S*-DABOs and analogs: in most cases the active compounds are characterized by a weak crystal structure, due to huge difficulty in purification, which is unachievable by crystallization without a preliminary chromatographic separation.

These first reports on 2-alkylthio-6-benzyl-4(3*H*)-pyrimidinones as non-nucleoside HIV-1 RT inhibitors opened a Pandora’s box for a plethora of different structural modifications of DABOs by several research teams. It was a sort of renaissance of 6-benzyl-2-thiouracils and their *S*-alkylated derivatives, which attracted much attention in the late 1940s/beginning of the 1950s as antithyroid compounds. Such a reincarnation of this well-known class of compounds in a brand-new one indicates that they may be undoubtedly recognized as privileged structures in drug design and discovery.

**Figure 4:**
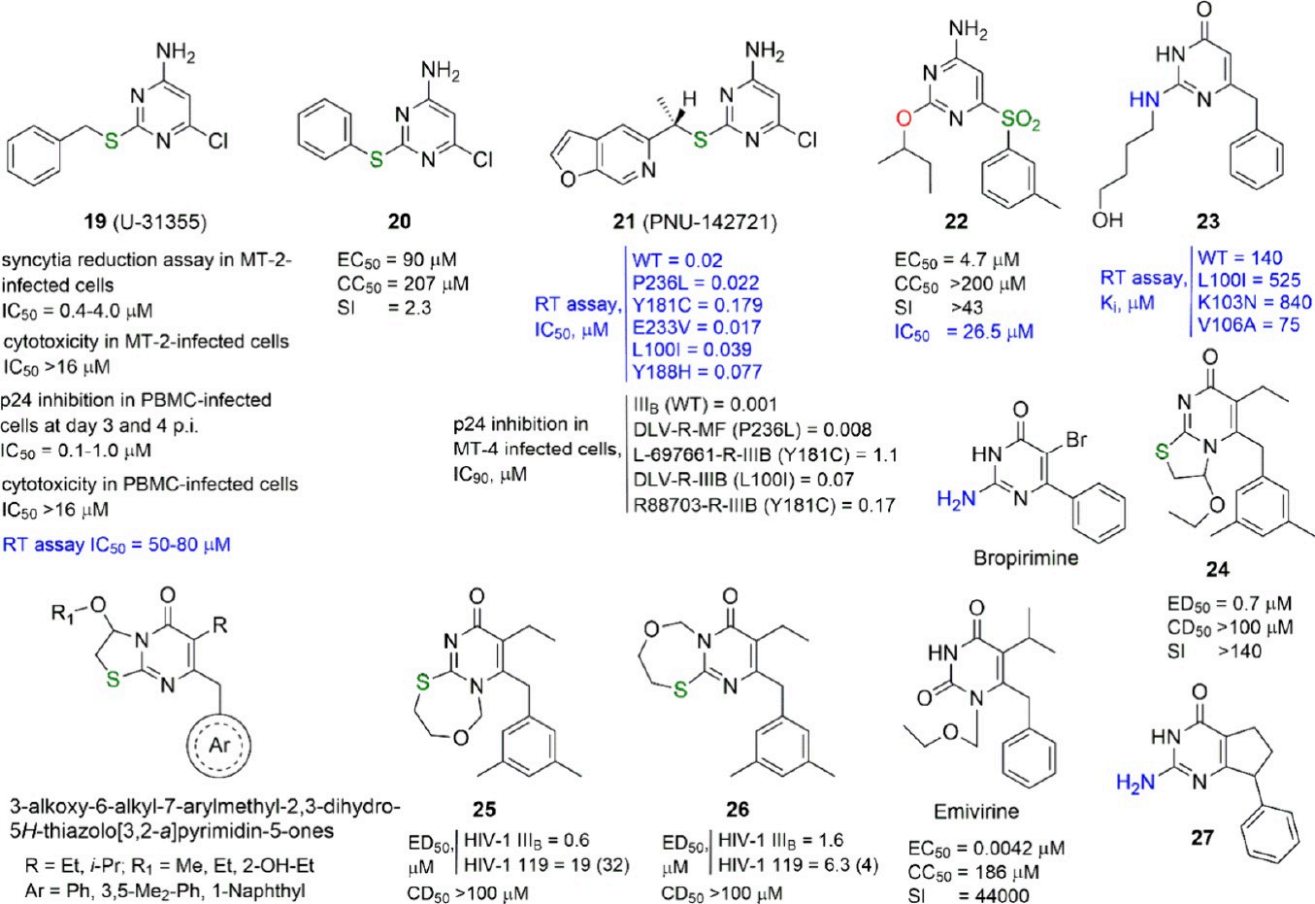
4-Aminopyrimidines and first examples of 2-amino-4(3*H*)-pyrimidinones. Annulated analogues or HEPTs/DABOs. For each compound, when available, the EC_50_ or ED_50_ (effective concentration able to induce 50% inhibition of HIV-1 cytopathic effect in cells), CC_50_ or CD_50_ (cytotoxic concentration able to reduce by 50% the viability of mock-infected cells), SI (selectivity index, ratio of CC_50_ or CD_50_ to EC_50_ or ED_50_), and IC_50_ (concentration of compound able to inhibit by 50% the HIV-1 RT activity) or *K*_i_ (in blue) values are reported. Fold-resistance is reported in parentheses when available.

In 1996, Althaus et al. of Pharmacia & Upjohn described the 4-amino-2-(benzylthio)-6-chloropyrimidine U31355 (**19**, Figure 4) as a non-nucleoside HIV-1 RT inhibitor,^19^ closely related to the virtually inactive analog **20** previously described by Massa et al.^12^ (Figure 4). Surprisingly, in the syncytia reduction assay using HIV-1-infected MT-2 cells, **19** showed an IC_50_ value between 0.4 and 4 μM together with a cytotoxicity of >16 μM.

Two papers published in 1998 by Pharmacia & Upjohn expanding the pioneering work of Althaus et al. claimed pyrimidine thioethers as a novel class of HIV-1 RT inhibitors, describing *S*-substituted 4-amino-6-chloro-2-mercaptopyrimidines containing benzyl groups or rigid olefin residues conjugated with tertiary amide fragments as the sulfur atom substituents.^20,21^ Since these compounds were related to those developed in 1994 by Massa et al.,^12^ their real novelty referred to the activity of these derivatives against HIV-1 strains resistant to BHAPs (bis-heteroaryl-piperazines), another class of anti-HIV-1 RT agents developed by the same company and including the FDA-approved delavirdine (Figure 1). The lead compound, the (−)-6-chloro-2-[(1-furo[2,3-*c*]pyridin-5-ylethyl)thio]-4-pyrimidinamine, PNU-142721 (**21**, Figure 4), proved to be potent in the nanomolar range against a panel of HIV-1 RT enzyme variants as well as against a panel of HIV-1 mutant strains in cells, including those that were BHAP-resistant.^20,21^ SAR studies showed that the presence of an electron-withdrawing substituent (preferably, Cl or CF_3_) at the C6 position of the pyrimidine ring is crucial for the anti-HIV-1 activity of these compounds, and the substitution of the sulfide linker with methylene, oxygen, or amino group, as well as its oxidation to sulfoxide or sulfone, negatively affects the antiviral activity.

In 2000, Costi and coauthors reported a series of new DABO-like compounds structurally related to those previously prepared by the same group (see **8** in Figure 3)^12^ and, at the same time, to Upjohn’s pyrimidine thioethers.^20,21^ Unfortunately, most of them turned out to be inactive as HIV-1 replication inhibitors. Those that did show antiviral activity did not show EC_50_ values below the single digit micromolar range (**22** in Figure 4) and were therefore clearly less potent than the DABO hits described above.^22^

Going back to 1998, Botta and co-workers reported a solid-phase synthesis of structurally diverse 2,6-disubstituted 4(3*H*)-pyrimidinones bearing an amino group at the C2 and/or N3 pyrimidine position.^23^ Among them, the 6-benzyl-*N*^2^-(4-hydroxybutyl)isocytosine **23** (Figure 4) attracted attention despite its low potency due to its ability to inhibit recombinant V106A mutant HIV-1 RT better than WT RT. Such a peculiar feature makes **23** a real precursor of the so-called *NH*- and *N*,*N*-DABOs, the *N*^2^-substituted or *N*^2^,*N*^2^-disubstituted 6-benzylisocytosines, developed later (see below). This branch of the whole DABO series also suggests a historical parallel with structurally related isocytosines, such as bropirimine (Figure 4), a known antiviral and anticancer agent.^24,25^ In other words, the appearance of *NH*- and *N*,*N*-DABOs represents another case of the renaissance of a privileged structure.

In parallel, the team of Pedersen attempted to hybridize *S*-DABO with HEPT compounds leading to the novel 2,3-dihydro-7*H*-thiazolo[3,2-*a*]pyrimidin-7-one **24** (Figure 4), which showed sub-micromolar potency in HIV-1 inhibition in cells.^26^ No data were reported about the behavior of such compounds in the enzymatic assays. Among these synthesized annulated analogues several intermediates, being true *S*-DABOs in their nature, showed HIV-1 replication inhibitory properties in the single-digit micromolar range. At the same time, the N1-cyclized regioisomers of the target compounds proved to be active against HIV-1, while their N3-cyclized analogues (general formula in Figure 4) did not, likely because of the presence of a steric hindrance at the N3 position. This confirmed data, previously reported by us,^14^ in which N3 methylation of an active *S*-DABO compound destroyed its antiviral activity.

A year later, another paper by the Pedersen group expanded their work on 2,3-dihydro-7*H*-thiazolo[3,2-*a*]pyrimidin-7-ones making a step forward to a ring expansion and describing some 7-(arylmethyl)-8-alkyl-2,3-dihydro-5*H*,9*H*-pyrimido[1,2-*c*][1,5,3]oxathiazepin-9-ones and 9-(arylmethyl)-8-alkyl-2,3-dihydro-5*H*,7*H*-pyrimido[1,2-*c*][1,5,3]oxathiazepin-7-ones (see **25** and **26**, Figure 4).^27^ These compounds displayed potencies similar to those of the previous thiazolo[3,2-*a*]pyrimidin-7-ones.

Another study, published by the Pedersen group in 2000,^28^ deserves some attention. During the synthesis and antiviral evaluations of some analogues of emivirine (Figure 4),^29^ a highly potent HEPT derivative, the 2-amino-7-phenyl-6,7-dihydro-5*H*-cyclopenta[*d*]-4(3*H*)-pyrimidinone **27** (Figure 4) was described, representing a sort of second *NH*-DABO described in literature. Unfortunately, it lacked anti-HIV-1 activity, possibly due to the absence of substitution on the exocyclic amino group.

## 3 2,6-Dihalobenzyl-*S*-DABOs and Analogues

**Figure 5:**
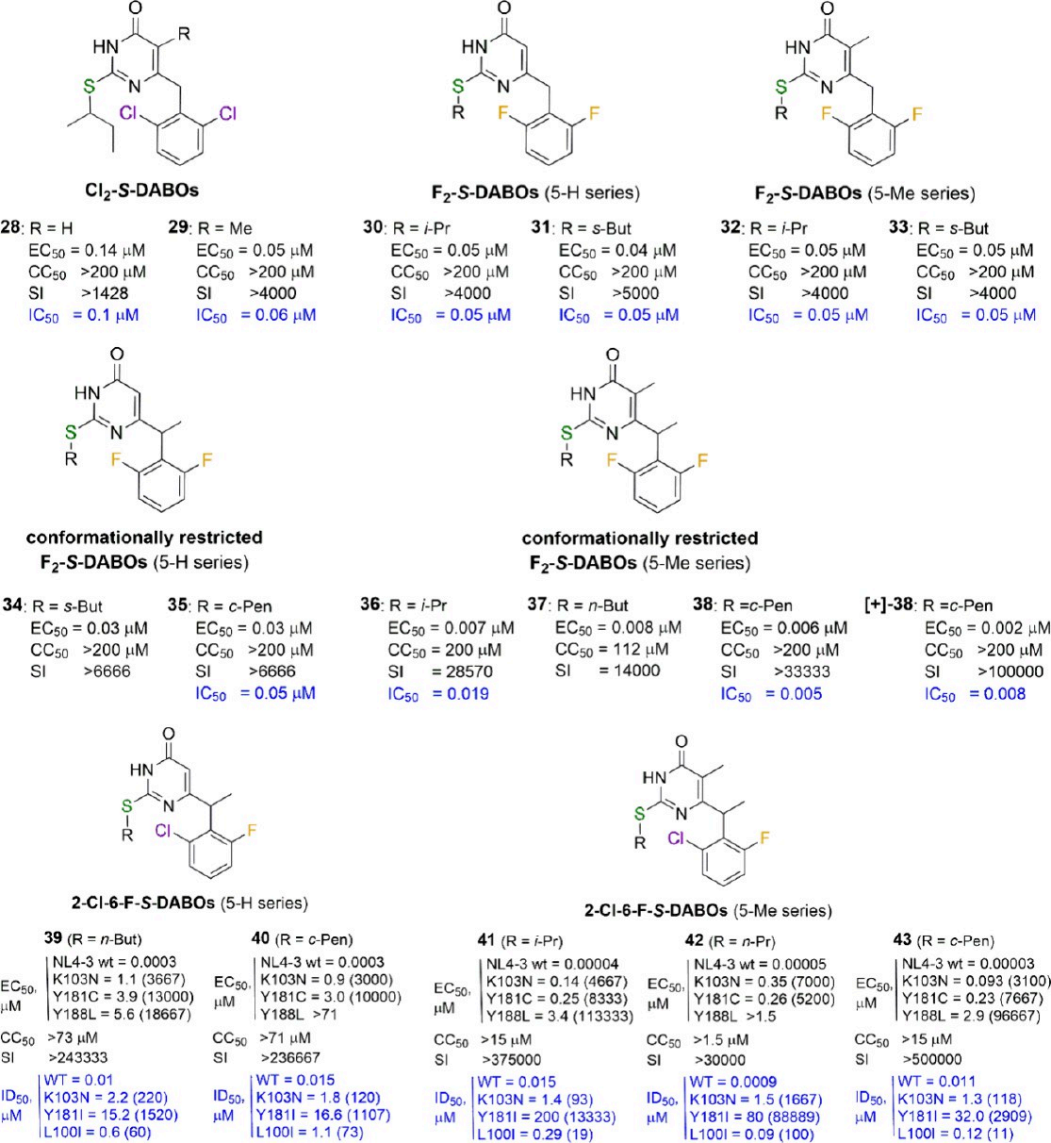
New 2,6-dihalo-*S*-DABOs and their conformationally restricted analogues. For each compound, the EC_50_ (effective concentration able to induce 50% inhibition of HIV-1 cytopathic effect in MT-4 cells), CC_50_ (cytotoxic concentration able to reduce by 50% the viability of mock-infected cells), SI (selectivity index, ratio of CC_50_/EC_50_), and IC_50_/ID_50_ (concentration of compound able to inhibit by 50% the HIV-1 RT activity, in blue) values are reported, when available. Fold-resistance is reported in brackets, when applicable.

The beginning of the new millennium was marked by the appearance of some papers on new DABOs and DABO-like compounds. During the year 2000, two major steps toward the optimization of *S*-DABOs were achieved with (i) the 2,6-dihalogenation of the benzene ring, reported by us in 1999^30^ and (ii) the insertion of a small alkyl group, like methyl or ethyl, at the benzyl α-position, reported by us in 2001.^31^ This substitution pattern proved to be an important milestone in all subsequent DABO history. Indeed, the introduction of one or preferably two electron-withdrawing groups at the *ortho*-position(s) of the *S*-DABO benzene nucleus remarkably pumps up the antiviral potency of the title compounds, with the (cyclo)alkyl chains at the sulfur atom at C2 having only modulatory effects. In general, the 2,6-difluoro substitution of the benzene ring (F_2_-*S*-DABOs) gave the best results, among the other replacements (compare the 2,6-dichloro analogues **28** and **29** with the 2,6-difluoro counterparts **30**–**33**, Figure 5). It is noteworthy that the 2-nitrobenzyl-substituted compounds turned out to be twice as potent as their corresponding 2-fluorinated counterparts. Nevertheless, 2,6-dinitrobenzyl or 2-fluoro-6-nitrobenzyl *S-*DABOs were not synthesized because of the very low water solubility shown by the 2-nitrobenzyl derivatives. Moreover, the nitro group is typically less promising for further drug design, as it may be reduced to the corresponding amine *in vivo*, leading to carcinogenic or mutagenic metabolites, as in the case with nitromethaqualone.^32^

Conformational restriction applied specifically to the thymine series of F_2_-*S*-DABOs through the insertion of a methyl group at the benzylic α-position increased the potency of the compounds by a further order of magnitude (**34**–**38**, Figure 5).^31^ Enantiomeric resolution of **38**’s racemate allowed to identify its [+] enantiomer as the eutomer (Figure 5; see below). Mutual van der Waals repulsion of C5 methyl and α-benzyl methyl groups forced the molecule into a conformation necessary for the optimal interaction with the biological target, thus minimizing the entropy losses during this process. In other words, the mode of interaction became less similar to a Koshland induced-fit model^33^ and more similar to a Fischer “key–lock” interaction.^34^ This simple and effective idea proved fruitful, leading to compounds active in the low nanomolar range and being 3 orders of magnitude more potent than the first DABOs.

In 2014, we systematically investigated the nonsymmetric 2-chloro-6-fluoro substitution of the benzene ring (2-Cl-6-F-*S*-DABOs).^35^ The most potent compounds of this series, bearing (i) a isopropyl, *n*-propyl, *n*-butyl, or cyclopentyl group bonded at the sulfur atom at C2 and (ii) the α-benzyl methyl (**39**, **40**) or (iii) the double C5/α-benzyl methyl (**41**–**43**) substitution, showed picomolar EC_50_ values toward WT HIV-1 but suffered from a remarkable loss of potency against K103N, Y181C, and, especially, Y188L mutant strains, with EC_50_ values in the sub-micromolar (K103N, Y181C) or single-digit micromolar (Y188L) range, indicating the dramatic effect of the latter mutation on their anti-HIV-1 activity (Figure 5). Enzymatic inhibitory data on WT and mutated RTs (K103N, Y181I, and L100I) were consistent with those of the cellular ones.

## 4 Methyl-Thio-Methyl (MTM)-*S*-DABOs and Their Congeners

**Figure 6:**
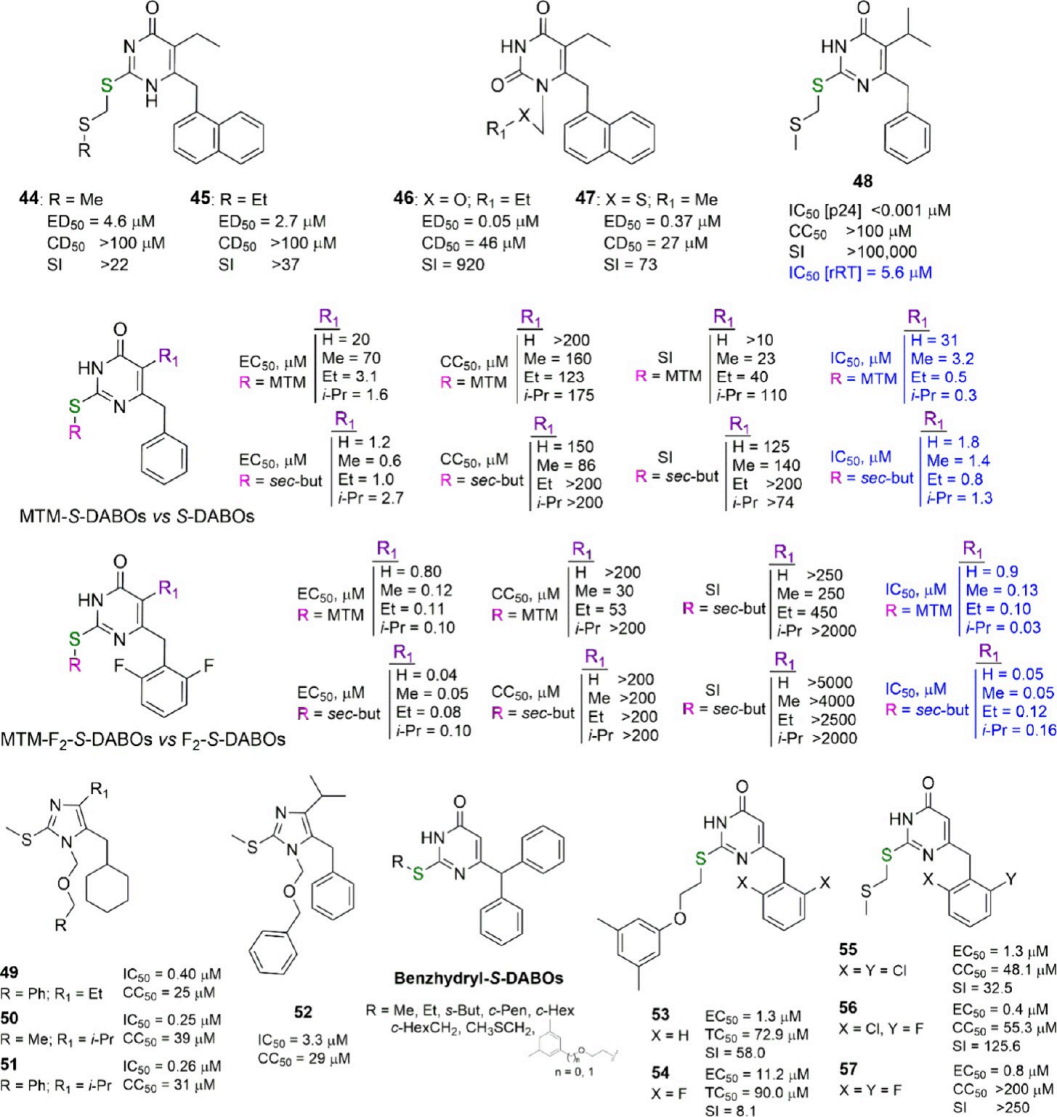
MTM- and MTM-like-*S*-DABOs, imidazole derivatives (“decarbonylated” *S*-DABOs), benzhydryl-*S*-DABOs, and 2-phenoxyethylthio-4(3*H*)-pyrimidinones. For each compound, EC_50_/ED_50_ (effective concentration able to induce 50% inhibition of HIV-1 cytopathic effect in cells), CC_50_/CD_50_/TC_50_ (cytotoxic concentration able to reduce by 50% the viability of mock-infected cells), SI (selectivity index, ratio of CC_50_/EC_50_), and IC_50_ (concentration of compound able to inhibit by 50% the HIV-1 RT activity, in blue) values are reported, when available.

In 1997, Pedersen and co-workers published a series of *S*-DABO and HEPT analogs (**44**, **45** and **46**, **47**, respectively) with a 1-naphthylmethyl substituent at C6 (similarly to DATNOs), an ethyl group at C5, and an (alkylthio)methyl chain at N1 (HEPT-like) or at the sulfur atom at C2 (*S*-DABO-like) and showing ED_50_ values in the single digit micromolar (DABO-like) or sub-micromolar (HEPT-like) range, with the first being less cytotoxic (Figure 6).^200^ This was the first paper reporting the (methylthio)methyl (MTM)-*S*-DABO analogues, in which an (alkylthio)alkyl substituent, similar to that present at the HEPT N1 position, was introduced at the sulfur atom at C2 in the DABO scaffold instead of the classical alkyl chain.

One year later, Uckun and coauthors issued two very similar articles dedicated to the breakthrough result obtained after the insertion in the *S*-DABO structure of the MTM group at the C2 sulfur atom (similar to that previously reported by Pedersen), the benzyl group at C6 and the isopropyl substituent at the pyrimidine C5 position (**48**, Figure 6).^36,37^ The unprecedented, sub-nanomolar cellular potency of **48** seems to be due to the different assay used to evaluate the anti-HIV-1 activity in cells, in this case the p24 production (IC_50_ [p24]). Despite this very high cellular potency, **48** showed single-digit micromolar activity in enzymatic assays (rRT), similar to the other reported *S*-DABOs.

In a paper published in 2000, we reported a comparative analysis of the data from Uckun and coauthors^36,37^ and the known active *S*-DABOs, by comparing the activity of *S*-*sec*-butyl and *S*-MTM 4-(3*H*)-pyrimidinones, all bearing benzyl or 2,6-difluorobenzyl groups at C6 (Figure 6).^38^ The C5 position carried a hydrogen, methyl, ethyl, or isopropyl group. The anti-HIV-1 potency of *S*-*sec*-butyl-substituted compounds was found to be higher than that of their MTM-substituted counterparts. As expected, 2,6-difluorobenzyl-substituted compounds were more effective than their benzyl analogues. Among the SAR data postulated by Uckun and co-workers, only one was confirmed: the activity of MTM-*S*-DABOs increases with stepwise transition from C5 hydrogen to C5 isopropyl, while the activity of *S*-*sec*-butyl substituted compounds decreased in the same way. Thus, a mixed DABO/HEPT SAR profile for the MTM-*S*-DABOs was proved.

In 2002, Pedersen and coauthors reported some ring-contracted or “decarbonylated” structural analogues of *S*-DABOs, some 2-(alkylthio)-4-benzyl-1*H*-imidazoles,^39^ also recognized as DABO–capravirine hybrids. Capravirine was a NNRTI developed by Pfizer but abandoned after phase II clinical trials.^40^ Since such compounds were inactive, the authors reasoned that “omitting” the carbonyl group in the *S*-DABO structure leads to the loss of the anti-HIV activity. Later it was shown that this conclusion was a little bit premature: indeed, in 2003 the Pedersen team reported a novel series of 1*H*-imidazoles with appropriate substitutions (a 1-[(benzyloxy)methyl] or 1-(ethoxymethyl) moiety at N1, a methylthio group at C2, an ethyl or isopropyl group at C4 and a cyclohexylmethyl residue at C5) as HIV-1 replication inhibitors (see **49**–**51**, Figure 6), showing sub-micromolar inhibitory potency against HIV-1-infected MT-4 cells.^41^ Only one among the C5-benzyl counterparts, **52**, exhibited comparable activity (at single-digit micromolar concentration), while the other benzylic analogues failed to inhibit HIV-1 replication. The main concept of this project was to introduce a nonplanar, more flexible cyclohexylmethyl fragment at the C5 position of the imidazole ring instead of the benzyl group. Such an idea was not really new, since the same approach was already applied by Tanaka and co-workers in 1999 in the structural optimization of emivirine.^42^ Yet, in their hands, the C6-cyclohexylthio group in TNK-6123 worked as well as the 3,5-dimethylbenzyl residue at C6 in GCA-186, leading to ∼30-fold greater inhibitory effect than emivirine against the clinically important Y181C and K103N mutant virus strains.^42^ This is in agreement with a seminal work of Lovering,^43^ declaring that the potential success of a drug candidate depends strongly on the number of sp^3^-hybridized atoms per molecule, together with the reduction of the number of planar and unsaturated fragments. In this respect, the replacement of the phenyl ring by its saturated analogue, performed by Pedersen and coauthors,^41^ was successful. This idea was later taken up by a group of scientists from Yunnan University and the Chinese Academy of Sciences and led to the development of new DABO [or better 3,4-dihydro-2-(arylalkyl)thio-6-(cyclohexylmethyl)-4-oxopyrimidine (DACO)] analogues (see below).^44−47^

Another paper by Pedersen and co-workers presented additional *S*-DABO derivatives bearing the 1-naphthyl (instead of 1-naphthylmethyl) substituent at the C6 position and MTM-like chains at the sulfur atom at C2: these compounds were endowed with low anti-HIV-1 potency,^48^ thus confirming what we had previously reported in 1997^14^ about the rejection of a single carbon linkage between pyrimidine and aromatic nuclei in the structure of *S*-DABOs.

In 2003, one of us reported several 6-benzhydryl-4(3*H*)pyrimidinones bearing alkyl-, arylalkyl-, or MTM-thio chains at C2 (Figure 6).^49^ The compounds showed two peculiar features, differentiating them from all the previously reported *S*-DABOs: (i) the introduction of an additional phenyl ring into the α-position of the benzylic portion of the molecule and (ii) the introduction of an aromatic residue into the alkyl chain inserted on the sulfur atom, illustrated by the 2-(3,5-dimethylphenoxy)- or 2-[(3,5-dimethylbenzyl)oxy]ethyl-substituted compounds and similar to an MTM-like substituent. Unfortunately, all the described compounds were inactive against HIV-1. The introduction of an aromatic ring into the alkyl chain linked to the sulfur atom at position 2 was also a feature of a few *S*-[2-(3,5-dimethylphenoxy)ethyl]-substituted *S*-DABOs, reported by Buckheit and coauthors in 2004.^50^ These compounds contained different substitution patterns at C5 and C6 of the pyrimidine nucleus and were totally inactive except for those bearing a hydrogen atom at C5 and a benzyl or 2,6-F_2_-benzyl at C6 (**53** and **54**, respectively, Figure 6), with the peculiarity that the 6-benzyl analogue **53** was approximately 8-times more potent than its 2,6-difluoro-substituted counterpart **54**, thus showing an inverse trend with respect to the SAR of known *S*-DABOs. Both compounds were remarkably cytotoxic as were their 6-benzhydryl-substituted predecessors.

Another short article in 2004 reported the synthesis of *S*-DABOs with the MTM chain at the C2 sulfur atom and 2,6-dichloro- and 2-chloro-6-fluorobenzyl at C6 (**55** and **56**, Figure 6).^51^ The 6-(2-chloro-6-fluorobenzyl)-2-MTM-4(3*H*)-pyrimidinone was found to be twice as potent as the previously reported^38^ 2,6-difluoro-substituted counterpart **57**, although it was more cytotoxic.

In 2010, the alkylation of (2,6-dihalobenzyl)-2-thiouracils and -thymines with iodomethane, (allylthio)methyl chloride, and (methylthio)methyl chloride in the K_2_CO_3_–DMF, NaOMe–MeOH, and KOH–EtOH systems was investigated. Unfortunately, only data for the 2-methylthio analogues were reported, with potencies in the sub-micromolar range for the WT HIV-1, and micromolar range (when active) for the HIV-1 tested mutants.^52^

In a subsequent investigation carried out in 2013, some 6-(2-thienylmethyl)-MTM-*S*-DABOs bearing (methylthio)methyl, (methylthio)ethyl, and (phenylthio)methyl groups at the sulfur atom at C2 were reported. These compounds were only tested as inhibitors of HIV-1 WT RT and showed very low activity with *K*_i_ values around 100–200 μM.^53^

## 5 DABO C5-Position: Introduction of Substituents Different from Small Alkyl Groups

**Figure 7:**
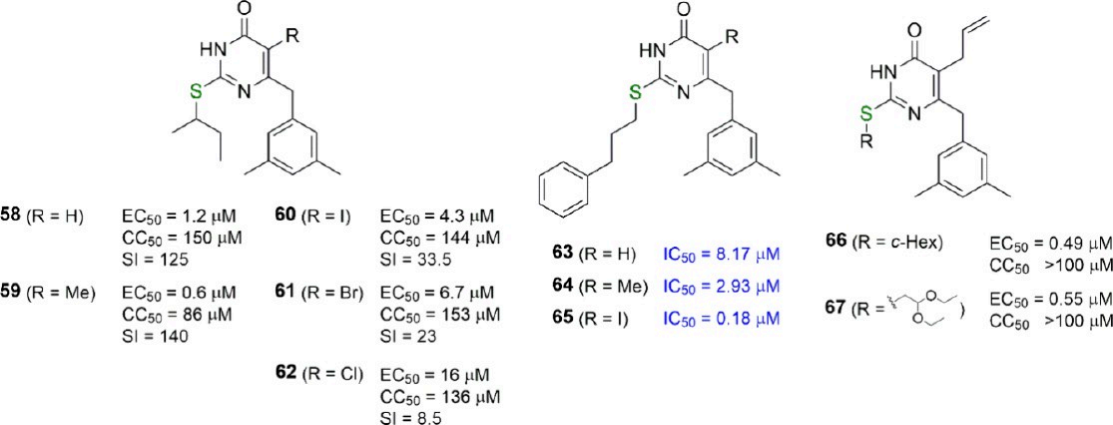
C5 substitution in *S*-DABOs. For each compound, the EC_50_ (effective concentration able to induce 50% inhibition of HIV-1 cytopathic effect in cells), CC_50_ (cytotoxic concentration able to reduce by 50% the viability of mock-infected cells), SI (selectivity index, ratio of CC_50_/EC_50_), and IC_50_ (concentration of compound able to inhibit by 50% the HIV-1 RT activity, in blue) values are reported, when available.

In 2001, we gained more insight into the SAR of DABO analogues by wiggling their structure. First, both the nature of the aryl group in the benzyl portion and the structure of the C5 pyrimidine ring substituent were investigated.^54^ As a result, the introduction of a charged or even polar (hydrophilic) substituent at C5 led to inactive or practically inactive compounds, thus indicating the need for the insertion of a lipophilic group at that position. Moreover, among Cl, Br, and I substituents at C5, iodine appeared to be the most preferable, likely because it is the least polar and the closest in size to the methyl group. Nevertheless, even 5-iodinated compounds were less potent than the unsubstituted ones or the corresponding 2-thiothymines (approximately 3 times) (compare **60**–**62** with **58**, **59**; Figure 7). Replacement of the phenyl ring in the benzylic moiety with bioisosteric pyridyl, thienyl, and 1*H*-pyrrol-1-yl rings led also to a drop of potency. The same effect was observed when bicyclic fragments, namely, 1*H*-indol-1- or -3-yl, thianaphthen-3-yl, or phthalimido groups, were introduced in place of the phenyl ring.

About 10 years later, Liu and co-workers further investigated the replacement of the substituent at the pyrimidine C5 position by inserting an iodine or bromine atom in a series of 6-benzyl-4(3*H*)-pyrimidinones carrying at C2 a (phenylalkyl)thio or (2-naphthylmethyl)thio residue.^55^ Surprisingly, they found the 5-iodo-2-(3-phenylpropyl)thio analogue to be more potent than its 5-H and 5-Me counterparts in enzymatic assays (against HIV-1 RT) (see **63**–**65**, Figure 7). Unfortunately, no data have been reported on the effects of the same compounds in HIV-1 infected cells or mutant RTs, and only an unsubstituted benzyl ring at C6 was utilized. However, the (arylalkyl)thio chains at C2 instead of the simple (linear, branched, or cyclic) alkylthio chains made the difference.

Again in 2001, the Pedersen group introduced a second benzyl group at the C5 position of the *S*-DABOs in addition to that inserted at C6, but all the compounds were devoid of anti-HIV-1 activity.^56^ In 2014, they also published a new series of *S*-substituted 5-allyl-6-(3,5-dimethylbenzyl)-2-mercapto-4(3*H*)-pyrimidinones with low micromolar/high sub-micromolar inhibitory potency against HIV-1 replication.^57^ The most potent derivatives were **66** and **67** (the latter was an intermediate for the synthesis of 6-allyl-5-(3,5-dimethylbenzyl)-3-ethoxy-2,3-dihydro-7*H*-thiazolo[3,2-*a*]pyrimidin-7-one and 6-allyl-7-(3,5-dimethylbenzyl)-3-ethoxy-2,3-dihydro-5*H*-thiazolo[3,2-*a*]pyrimidin-5-one, all devoid of anti-HIV-1 activity) bearing a cyclohexylthio and a (2,2-diethoxyethyl)thio portion, respectively, at C2 (Figure 7).

## 6 DATA and DAPY Derivatives

**Figure 8:**
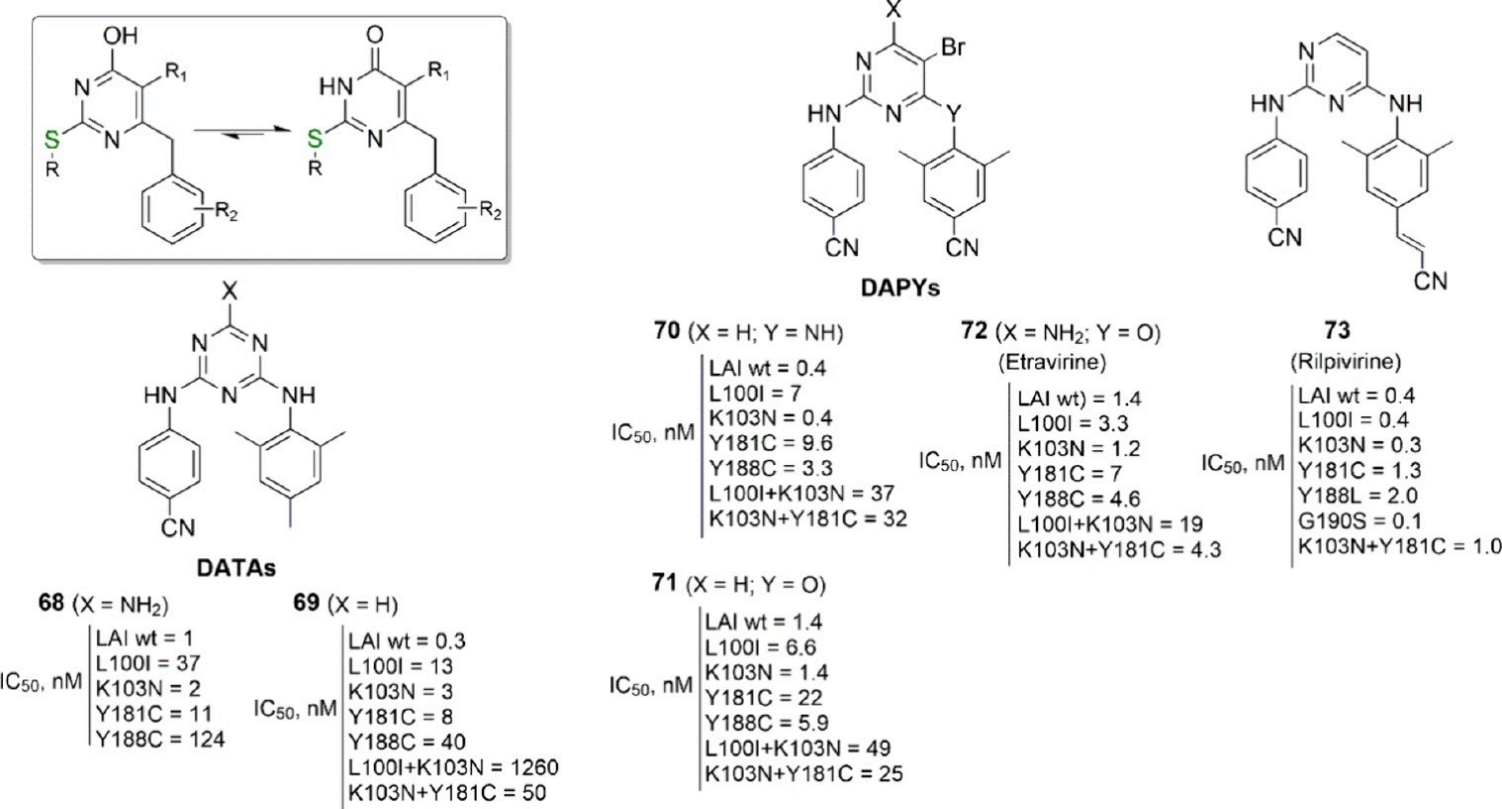
Tautomerism for 4(3*H*)-pyrimidinones. DATA and DAPY compounds from the Janssen laboratories, including the clinically approved etravirine and rilpivirine. The published IC_50_ (effective concentration able to induce 50% inhibition of HIV-1 cytopathic effect in cells) values are reported.

In 2001, Ludovici and co-workers from Janssen reported two novel classes of HIV-1 replication inhibitors of non-nucleoside origin related to DABOs: the diaryltriazines (or DATAs) and the diarylpyrimidines (or DAPYs), bearing a 1,3,5-triazine or a pyrimidine nucleus as a central core, and the substitution with a *p*-cyanoaniline fragment at C2, an amino or H group at C4, and a benzyl, phenylthio, phenoxy, or anilino group at C6 bearing different substituents (mainly Cl, Me, Br, NO_2_) at 2′,4′,6′ positions (Figure 8).^58,59^ These compounds appeared to be very close in structure to the DABO series but with two substantial differences. First, the pyrimidine/triazine ring was “locked” as a major aromatic tautomer, while in the case of the known DABOs the aromatic tautomer was the minor one. Second, the substituent at C2 always contained an aromatic ring (an aniline moiety) instead of an aliphatic group. Most of the DATAs featuring an amino substituent at C4 (see, for instance, **68** in Figure 8) displayed low/single-digit nanomolar potency against the WT HIV-1 LAI strain and slightly reduced potency against a panel of mutant strains in MT-4 cells, and DATAs unsubstituted at C4 (see **69** in Figure 8) were the most potent compounds of the whole series, with potency at the sub-nanomolar level against WT LAI HIV-1 and low/single-digit nanomolar potency against the mutant HIV-1 strains. However, among DATA compounds, **69** was completely inactive against the double L100I + K103N mutant (Figure 8).^58^

To elicit low nanomolar potency against the double mutant strains L100I + K103N and K103N + Y181C, it was necessary to replace the triazine with the pyrimidine nucleus (DAPYs, closer analogues of DABOs than DATAs) and to decorate the pyrimidine with (i) the 4-cyanoanilino at C2, (ii) the H or amino group at C4, (iii) the bromo atom at C5, and (iv) the 2,6-dimethyl-4-cyanoanilino or -phenoxy group at C6 (see **70**–**72** in Figure 8).^59^

From all this efforts, two DAPY compounds, etravirine (compound **72**, Figure 8) and the 2-[(4-cyanophenyl)amino]-6-{4-[(2-cyanovinyl)-2,6-dimethylphenyl]amino}pyrimidine rilpivirine **73** (Figure 8), were approved for the clinical use against HIV-1 infection in 2008 and 2011, respectively (see also Figure 1).^4^ However, they suffered from some toxicity issues such as cardiotoxicity and the ability to cause myocardial infarction (especially in the case of etravirine).^60^ From this point of view, maybe the classical DABO structure seems to be more promising because it is devoid of the potential toxicophore, a pyrimidine nucleus linked at the C2 and C6 positions to two benzene rings via the single-atom bridges. In this respect, the work of Ritchie and coauthors,^61,62^ indicating that the multiplication of planar aromatic rings per small molecule (3 being a critical point) reduces the chances of clinical success due to the hidden toxicity, is in absolute agreement with the above-mentioned evidence.

## 7 2-Aryl/Arylalkyl-*S*-DABOs

**Figure 9:**
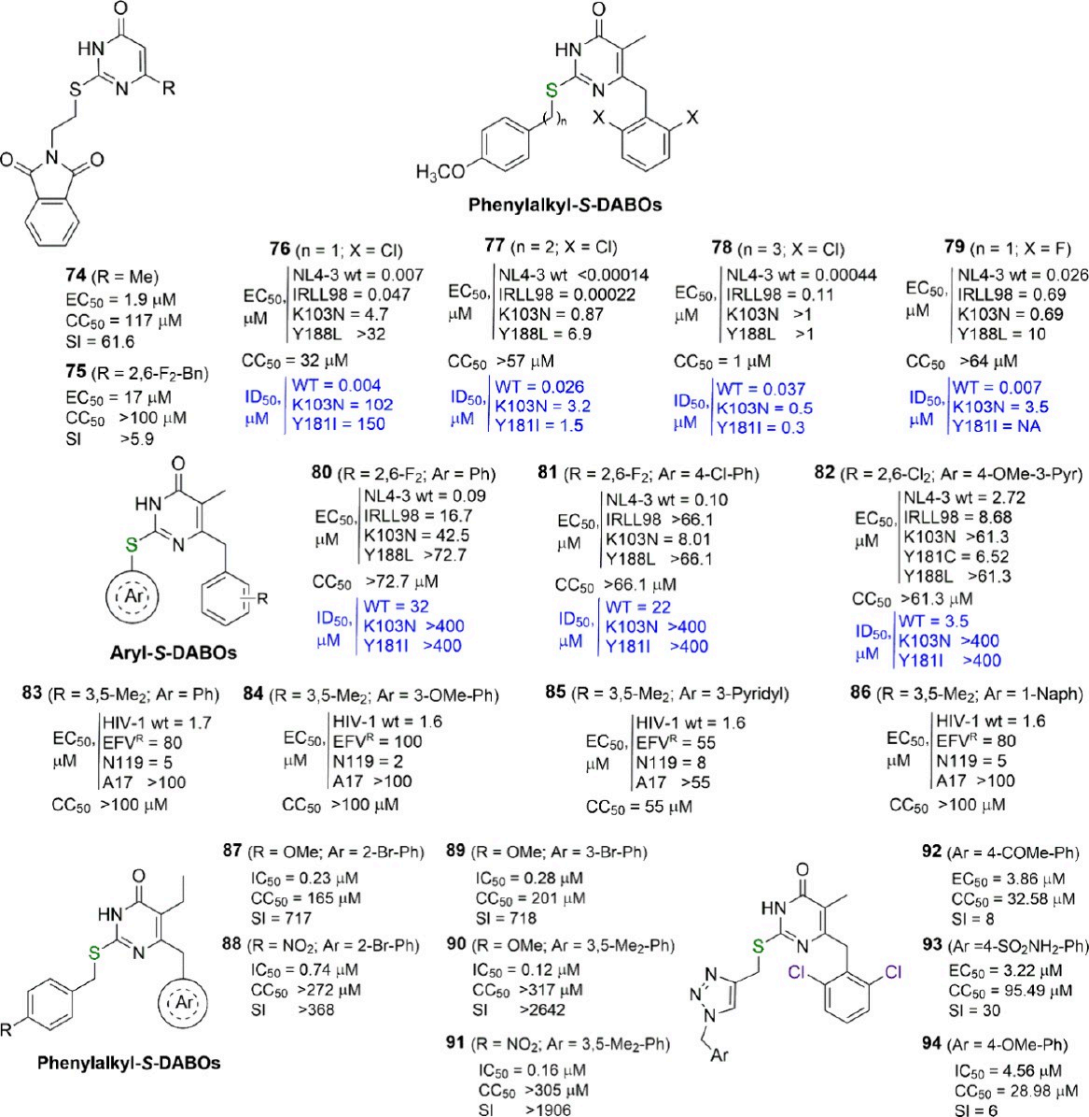
Arylalkyl-*S*-DABOs and related analogues, aryl-*S*-DABOs. For each compound, the EC_50_/IC_50_ (effective concentration able to induce 50% inhibition of HIV-1 cytopathic effect in cells), CC_50_ (cytotoxic concentration able to reduce by 50% the viability of mock-infected cells), SI (selectivity index, ratio of CC_50_ to EC_50_ or IC_50_), and ID_50_ (concentration of compound able to inhibit by 50% the HIV-1 RT activity, in blue) values are reported, when available. IRLL98 = clinical isolate bearing the K101Q, Y181C, and G190A mutations conferring resistance to nevirapine, delavirdine, and efavirenz. EFV^R^ = K103R + V179D + P225H mutant. N119 = Y181C mutant. A17 = K103 + Y181 mutant.

An interesting brief paper in 2005 described some 2-[(2-phthalimidoethyl)thio]-4(3*H*)-pyrimidinones carrying different substituents at C5 and C6 positions of the pyrimidine nucleus, prepared as prodrugs which may be converted to the corresponding 2-[(2-aminoethyl)thio]-4(3*H*)-pyrimidinones via hydrolytic cleavage of the phthalimide group *in vivo*. This did not occur, but the SAR resulting from the synthesized compounds were in conflict with those obtained with all the previously reported *S*-DABOs, because their anti-HIV-1 activity was lost with double methylation at C5/C6 (see **74**, Figure 9), and the C6-methyl substitution (with no substituent at C5) resulted in a potency approximately 9-times higher than its 6-(2,6-difluorobenzyl) counterpart (**75**, Figure 9).^63^ Thus, these compounds seemed to have a different binding mode or even mechanism of antiviral activity compared to all DABOs reported earlier. Two years later, another paper of this kind was published by Nielsen and coauthors.^64^ The main and peculiar features of the title compounds were the introduction of 2-(pyrrolidin-1-yl)ethyl or 2-(piperidin-1-yl)ethyl residue at the sulfur atom and a 4-chlorobenzyl group at C6, but the resulting molecules were devoid of any anti-HIV-1 activity.

The strategy of introducing phenylalkyl substituents at the C2 position of the *S*-DABO pyrimidine ring was resumed in 2005 by the Botta research team, who used parallel solution-phase synthesis to produce *S*-(ω-arylalkyl)thio-4(3*H*)-pyrimidinones and their products of *S*-oxidation, carrying 2,6-dichloro- or 2,6-difluorobenzyl group at C6.^65,66^ Several of the reported compounds showed remarkable potency, being active against HIV-1 at nanomolar or even sub-nanomolar concentrations in cellular assays (see **76**–**79**, Figure 9). Noteworthy, among the *S*-substituents, the 4-methoxyphenethyl group yielded the most potent derivatives, while elongation or shortening of the ethylene linker led to less active and/or more toxic compounds. Surprisingly, 2,6-dichlorobenzyl-substituted derivatives were equally effective to or even more effective than their 2,6-difluorobenzyl analogues. Finally, both bioisosteric sulfides and sulfones proved to be active in enzymatic assays, while in cellular assays only the sulfides retained their activity. The reason for such a peculiarity may be the solvolytic stability of sulfides, while sulfones are easily hydrolyzed in cell culture medium. The parallel solution-phase synthesis was used by the same research group to produce some 4-(*N*,*N*-dialkylamino)-2-methylsulfonyl-6-vinylpyrimidines, still endowed with anti-HIV-1 activity but only at micromolar level.^67^

Different from the 2-(phenylalkyl)thio-DABOs, the 2-phenylthio-DABOs synthesized by the same team by microwave-assisted copper-mediated arylation with arylboronic acids were only moderately active as HIV-1 replication inhibitors and generally much less potent or totally inactive against mutant strains and both WT and mutant RTs (**80**–**82**, Figure 9).^68^ In this case, even 4-methoxyphenylthio or similar substitutions did not serve to confer inhibitory potency. In contrast to the DAPY etravirine, the introduction of the 4-cyanophenylthio residue at C2 (bioisosteric replacement for the 4-cyanoaniline moiety of etravirine) appeared to be a nonoptimal structural pattern, thus suggesting a different mode of interaction with the biological target.

The modest results obtained with these compounds did not prevent Pedersen and coauthors from following the same direction and preparing a new series of aryl-*S*-DABOs, synthesized via a classical Ullman procedure and carrying different substituents (*meta*-substituted phenylthio, heteroarylthio) at C2 and 3,5-dimethylbenzyl group at C6 (**83**–**86**, Figure 9).^69^ All of the screened compounds were less potent than emivirine against the WT HIV-1 strain, but several of them appeared to be remarkably potent against the clinically relevant Y181C mutant strain N119. Another brief series of novel *S*-DABOs, reported by the same research team and apparently prepared just as byproducts in the synthesis of emivirine analogues containing a 3-(allyloxy)benzyl group at pyrimidine C6 and capable of bind covalently to HIV-1 RT, resulted devoid of any anti-HIV activity.^70^

In 2009, the group of Chen took up the arylalkyl-*S*-DABOs previously described by Botta and collaborators and explored (i) various arylmethyl substitutions at C6, using 2-, 3-, or 4-bromobenzyl, 3,5-dimethylbenzyl, 3,5-di(trifluoromethyl)benzyl, or 1-naphthylmethyl moiety, (ii) a methyl or ethyl group at C5, and (iii) a fixed 4-substituted benzyl group at C2.^71^ Among the substituents newly introduced at C6, the 2- and 3-bromobenzyl and the 3,5-dimethylbenzyl groups appeared to be the most promising ones (**87**–**91**, Figure 9), with a SAR profile including both emivirine and DABO analogue elements, i.e., the 2-halo- as well as the 3,5-dimethylbenzyl substitution at C6 and the ethyl group at C5 allowed the highest potency. All of the compounds were inactive against the HIV-2_ROD_ strain. No data were provided on the activity against either HIV-1 mutant strains or WT and mutant RTs.

A series of 2-[(1-benzyl-1,2,3-triazol-4-yl)methylthio]-4(3*H*)-pyrimidinones carrying a 2,6-dichlorobenzyl or a 1-naphthylmethyl moiety at C6 were reported in 2015 by Liu, Zhan and co-workers as a variation of the 2-(arylalkyl)-*S*-DABOs. However, the most potent of such compounds showed EC_50_ values in the low micromolar range against HIV-1 infected cells, no activity against HIV-2 and the HIV-1 mutant strain RES056, and some cytotoxicity (**92**–**94**, Figure 9).^72^

## 8 2-Phenacyl-*S*-DABOs and Analogues

**Figure 10:**
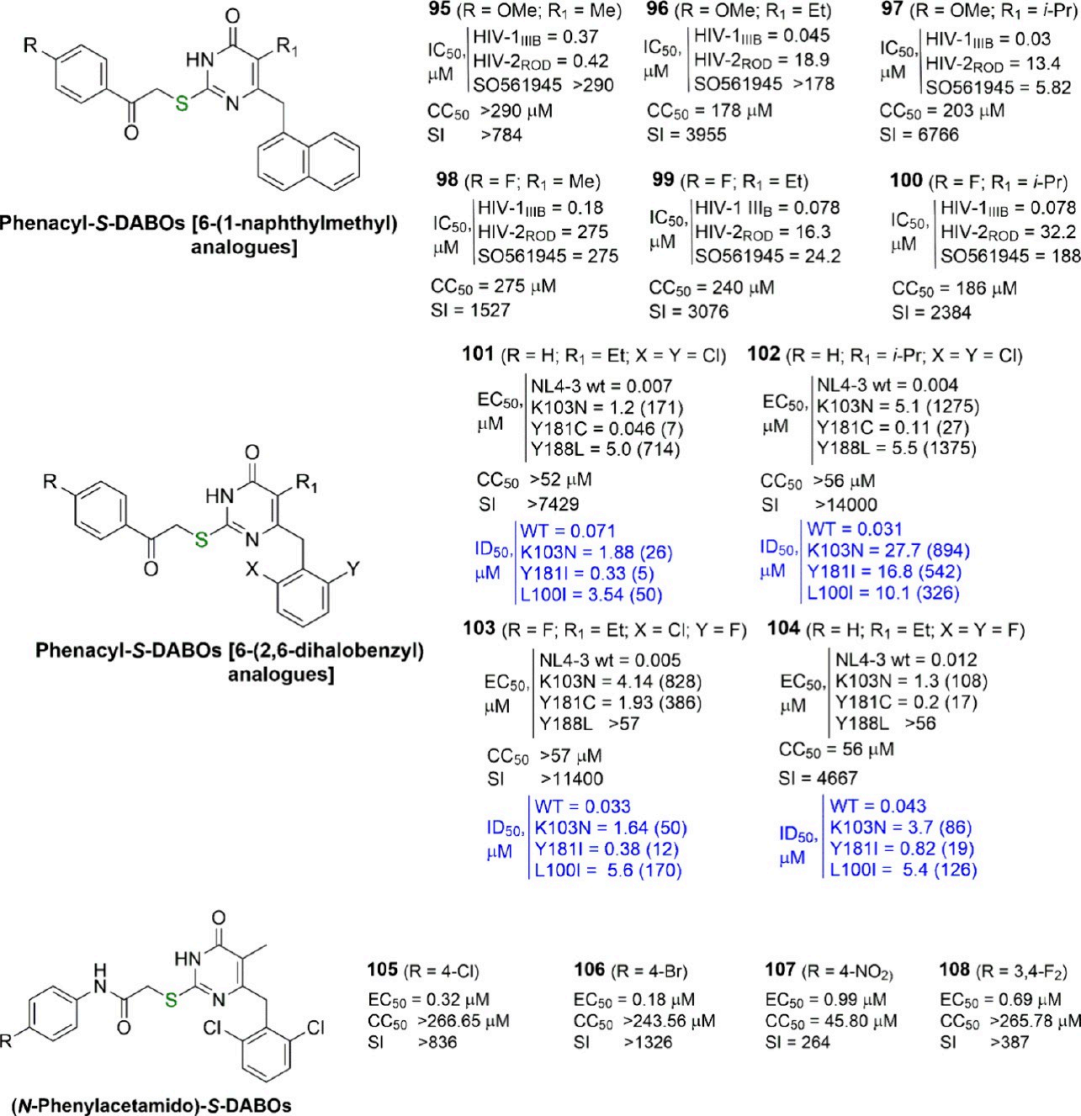
Phenacyl-*S*-DABOs and analogues. EC_50_ (effective concentration able to induce 50% inhibition of HIV-1 cytopathic effect in MT-4 cells), CC_50_ (cytotoxic concentration able to reduce by 50% the viability of mock-infected cells), SI (selectivity index, ratio of CC_50_/EC_50_), and ID_50_ (concentration of compound able to inhibit by 50% the HIV-1 RT activity, in blue) are reported when available. Fold-resistance is reported in brackets.

The “aromatic–aliphatic” pattern in the sulfur atom decoration of *S*-DABOs was also investigated by Chen et al. In 2004, they reported the new *S*-phenacyl *S*-DABOs together with their close structural analogues in two very similar papers.^73,74^ These scientists proposed a crucial role of hydrogen bond formation between the carbonyl oxygen in a sulfur atom side chain and the *NH*-group of Tyr318 residue in the RT binding pocket for the tight substrate–enzyme binding. The 4-methoxyphenacyl- and 4-fluorophenacyl-substituted compounds were the most potent, and their potency increased with a sequential transition from methyl to isopropyl at the pyrimidine C5 position (**95**–**100**, Figure 10). More intriguingly, several of these compounds also inhibited HIV-2 replication, an unprecedented feature for the DABO derivatives, similar to SJ-3366, an emivirine analog developed by Buckheit and coauthors.^75^ Moreover, some of them showed micromolar activity against SO561945, an HIV-1_IIIB_ strain with typical NNRTI-selected mutations in the RT (K103N and Y181C). The SAR profile exhibited by these compounds is similar to those of emivirine and MTM-*S*-DABOs, described previously. In this respect, it is surprising that 1-naphthylmethyl was preferred as a substituent at the C6 position, as we had already shown that the 2,6-difluorobenzyl moiety at C6 performs better for *S*-DABOs,^30^ whereas the 3,5-dimethylbenzyl group at C6 is preferred for emivirine analogues.^42^ Additional compounds synthesized by the same research group and characterized by the replacement of the C6 1-naphthylmethyl moiety with a 1-naphthylthio/phenylthio ring, more similar to HEPT, were less potent against HIV-1 and inactive against HIV-2.^76^

In 2008, we completed the research on 2-phenacyl-*S*-DABOs by simple introduction of the more favorable 2,6-dihalophenyl moiety instead of 1-naphthyl in the pyrimidine C6-methylene position.^77^ The new 2,6-dihalobenzyl-2-phenacyl-*S*-DABOs were potent at nanomolar or sub-nanomolar level against WT HIV-1 and often retained their activity against the clinically relevant mutants K103N, Y181C, and Y188L. Also, in enzymatic assays, many of them retained their potency toward K103N, Y181I, and L100I HIV-1 RT mutant forms. In general, these compounds exhibited a SAR profile more similar to that of emivirine analogues rather than that of classical *S*-DABOs, with (i) ethyl and isopropyl substituents at C5 being better than hydrogen and methyl and (ii) 2-chloro-6-fluoro- and 2,6-dichlorobenzyl groups at C6 often being better than the 2,6-difluorobenzyl ones (**101**–**104**, Figure 10).

In the same year a related work on 6-benzyl- and 6-(1-naphthylmethyl)-substituted 4(3*H*)-pyrimidinones bearing (hetero)aroylmethylthio and simple heteroarylthio substituents at C2 was published by Zheng, He, and their co-workers.^78^ The main feature of this project was the introduction of an electron-rich furan-2-yl or thiophen-2-yl instead of a benzene ring at the C2 sulfur atom. Unfortunately, this attempt was not very fruitful, because the phenacyl counterparts with the ethyl or isopropyl at C5 were more potent anyway, with the C6-benzyl being more effective than the 1-naphthylmethyl analogues. No data on HIV-1 mutant strains, as well as on mutant RTs were reported for these compounds.

In 2009 and 2011, the group of Liu reported a novel series of 6-(2,6-dichlorobenzyl)-4(3*H*)-pyrimidinones carrying an (*N*-phenylacetamido)thio substituent at C2 and a hydrogen atom or a methyl or ethyl group at C5.^79,80^ Despite these compounds being retained as structural analogues of 2-phenacyl-*S*-DABOs, unfortunately, the introduction of the aniline instead of the phenyl fragment in the side chain appeared to be detrimental for the antiviral activity. The 5-H derivatives were typically more toxic than the 5-methyl ones, and among the latter, the 4-chloro-, 4-bromo-, 4-nitro-, and 3,4-difluoroanilino analogues showed the highest potency and selectivity against HIV-1_IIIB_ (**105**–**108**, Figure 10). All the compounds were inactive against the HIV-2_ROD_ strain. No data were provided on the activity against either HIV-1 mutant strains or WT or mutant RTs.

## 9 *NH*- and *N*,*N*-DABOs

**Figure 11:**
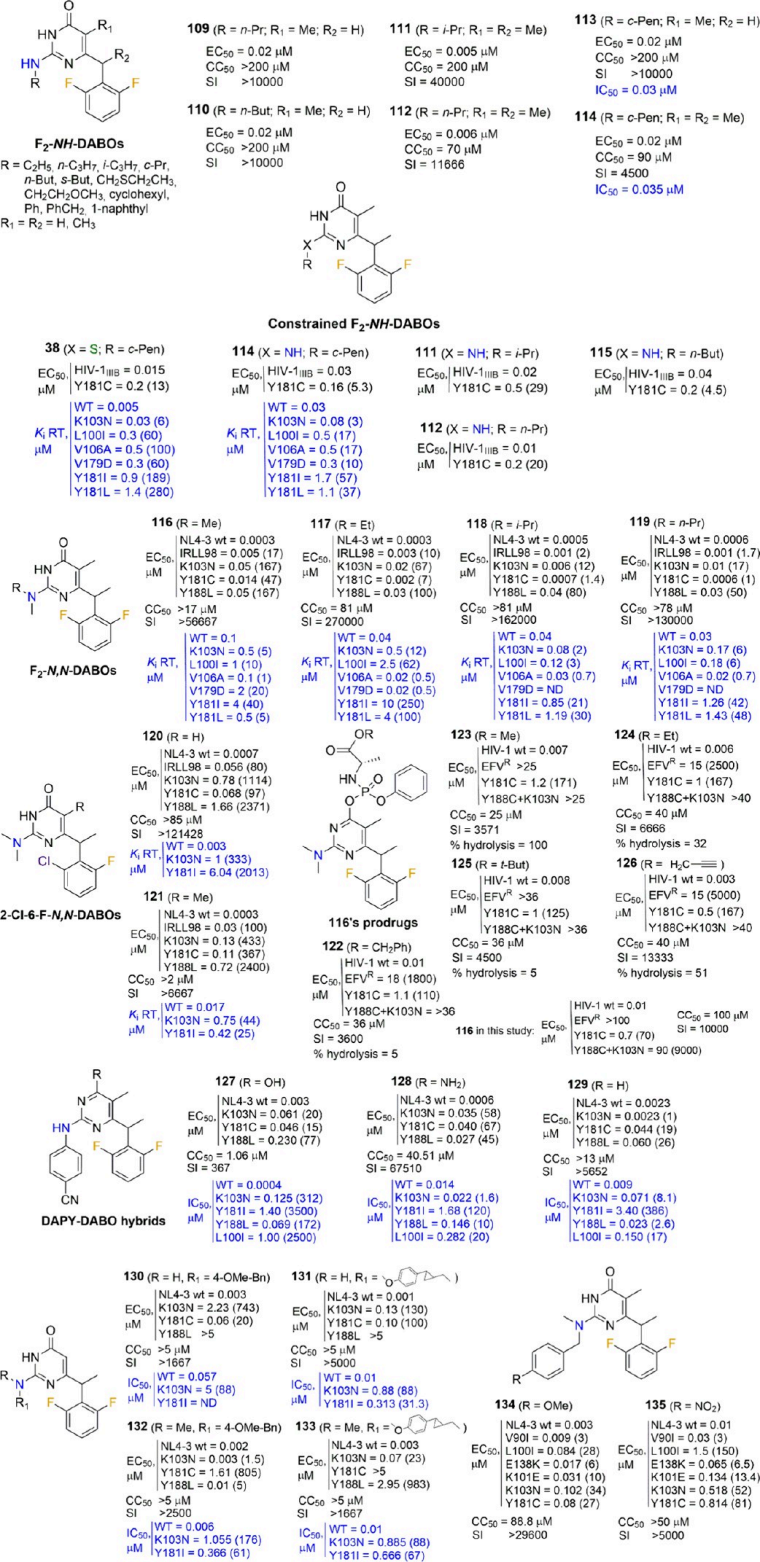
F_2_-*NH*-DABOs. Comparison between F_2_-*S*- and F_2_-*NH*-DABOs, F_2_-*N*,*N*-DABOs, and 2-Cl-6-F-*N*,*N*-DABOs. Phosphoramidate prodrugs of compound **116**. DAPY-DABO hybrids. EC_50_ (effective concentration able to induce 50% inhibition of HIV-1 cytopathic effect in cells), CC_50_ (cytotoxic concentration able to reduce by 50% the viability of mock-infected cells), SI (selectivity index, ratio of CC_50_/EC_50_), and IC_50_ (concentration of compound able to inhibit by 50% the HIV-1 rRT activity, in blue) are reported when available. Fold-resistance is reported in brackets.

In 2004–2005, we introduced a major novelty to the DABO family by describing for the first time some 2-alkylamino/arylamino-6-[1-(2,6-difluorophenyl)alkyl]-4(3*H*)-pyrimidinones as highly active anti-HIV-1 agents (F_2_-*NH*-DABOs).^81,82^ A large number of different 6-(2,6-difluorobenzyl)isocytosines carrying different substituents at the exocyclic nitrogen atom at C2 were prepared and screened for their antiviral activity and cytotoxic properties. These compounds exhibited slightly lower potency than the corresponding sulfur-containing analogues, likely due to the unwanted hydrogen bond formed by an exocyclic *NH*-group, with the *n*-propyl- and isopropyl-amino chains being the best substituent at C2 in the 5-methyl/6-(α-methyl)benzyl constrained form (**109**–**115**, Figure 11). Several 2-anilino-4(3*H*)-pyrimidinones were also prepared, related to the previously reported DAPYs, with, at the best, similar potency to the cyclohexylamino counterparts. As an added value, F_2_-*NH*-DABOs seem to lose less potency than their sulfur analogues against resistant mutants, at both the enzyme and cellular (Y181C) level (compare **38** and **114** in Figure 11).

Further development of the F_2_-*NH*-DABO derivatives led us to describe a series of 2-(*N*,*N*-disubstituted) amino-6-2,6-difluorobenzyl-4(3*H*)pyrimidinones (F_2_-*N*,*N*-DABOs),^83^ characterized by the ability to block an unwanted hydrogen bond produced by the monosubstituted exocyclic nitrogen atom of the F_2_-*NH*-DABOs. In this respect, the most advantageous substitution patterns were (i) the *N*,*N*-dimethyl, *N*-methyl-*N*-ethyl, and mainly the *N*-methyl-*N*-isopropyl and -*N*-*n*-propyl substitutions at C2, joined to (ii) the 6-[1-(2,6-difluorophenyl)ethyl]-5-methyl-4(3*H*)-pyrimidinone core, which guaranteed the constrained shape of DABOs (**116**–**119**, Figure 11) and led to sub-nanomolar potency for the antiviral activity without any remarkable change in their cytotoxicity. Each of these hit compounds possessed its own peculiarities. The 2-(*N*,*N*-dimethylamino)-4(3*H*)-pyrimidinone **116** turned out to be the most convenient in terms of synthesis and purification but less promising than the other leads from the SAR point of view, due to its scant water solubility. On the other hand, the 2-[isopropyl(methyl)amino]-4(3*H*)-pyrimidinone **118** and its *n*-propyl analogue **119** appeared to be the most potent derivatives, with respect to both HIV-1 WT and its clinically relevant mutant strains.^84^ Also, **118** and **119** are the analogues that retained the greatest potency against the mutant RTs compared to the WT RT (Figure 11). Moreover, **119** inhibited HIV-2 replication also, albeit at much higher concentrations (EC_50_^HIV-2_ROD_^ = 8.6 μM).^85^ Unfortunately, these compounds were the most difficult to prepare and to purify. An extremely poor crystallizability of **119**, together with a very low yielding aminolysis procedure for the synthesis of its isopropyl analogue **118** (due to the use of a branched amine) appeared to be critical to the success of their large-scale preparation.

The 2-chloro-6-fluorobenzyl analog of compound **116** was also prepared, together with its 5-H derivative, with the aim to improve its RT association rate and antiviral potency.^86^ The two constrained analogues **121** and **120**, respectively, were remarkably more potent than **116** against WT RT and, for the closest analog **121**, against the mutant Y181I RT (Figure 11). More interestingly, they showed improved association rates toward HIV-1 RT WT and two mutated forms (K103N and Y181I), with a preferential association with mutated forms of HIV-1 RT either in the unbound state (free enzyme) or in complex with the nucleic acid substrate (binary complex). In contrast, their interaction with the mutated enzymes is stabilized by the nucleotide binding to the enzyme, resulting in very low dissociation rates of the inhibitor from the viral RT. Unfortunately, in cellular assays **120** and **121** were as potent or less potent than **116** against both HIV-1 WT and, especially, its clinically relevant mutant strains.

Compound **116** was taken as a model for the development of several series of analogues by others. In 2007, the Pedersen group reported some amino-DABOs, bearing an *N*,*N*-dimethylamino group at C2, a methyl or ethyl at C5, and a 3-bromobenzyl group at C6, as intermediates in a wider work for the synthesis and biological activity evaluation of emivirine analogues with alkynyl-substituted C6-benzyl groups.^87^ Among these compounds, only one was tested, showing poor results as an HIV-1 replication inhibitor. The same authors also reported 1,3-phenylene bis-aminoDABOs, in which two 2-(*N*,*N*-dimethylamino)- or 2-piperidino-5-ethyl-4(3*H*)-pyrimidinone residues were linked to the α,α′-positions of *meta*-xylene, via pyrimidine C6 carbon atoms.^88^ The main idea of this nontrivial work was to find out whether an additional part of the modified nucleobase, attached to the benzene ring of the amino-DABO scaffold, could lead to better inhibitory activity of the target compounds against both HIV-1 WT and its Y181C and Y181C + K103N mutant strains. Unfortunately, this mixed approach led to inactive compounds.

Other structural analogues of **116** were investigated by the same group in 2010 and in 2016 with variation at the α-benzylic position (see below).^89,90^ In 2016, the Pedersen group prepared a series of *O*^4^-phosphoramidate prodrugs of **116**, which were comparable or better in their potency against WT HIV-1 but more cytotoxic and with different percentage of hydrolysis in RPMI medium after 4 day incubation (**122**–**126**, Figure 11).^91^ Apparently, the main purpose of this work was to escape the existing patents on DABO analogues, in which the presence of an unmodified NHCO group at the 3,4-position was a peculiar feature. It is difficult to say whether such prodrugs make any sense from this point of view, but it was clear that due to the structure of the *O*^4^ substituents, they may be endowed with several different hidden toxicity issues in animal model experiments. Even the release of a phenol molecule during the prodrug hydrolytic cleavage was already a negative feature. Moreover, considering the structural similarity of such prodrugs to known acetylcholinesterase inhibitors (for instance, diazinon^92^ with a similar structure), they may be endowed with human toxicity.

An important feature of derivative **116** was its efficacy as a topical microbicide. The availability of an effective vaginal microbicide would be a major step in reducing the sexual spread and transmission of HIV. Moreover, it could also enable women to protect themselves against infection. Among the RT inhibitors, chronic treatment with the nucleoside derivatives (didanosine, zalcitabine, stavudine, and lamivudine) simply delays the viral breakthrough with respect to infected, untreated controls (virustatic action). In contrast, NNRTIs (emivirine, α-APA, nevirapine, efavirenz, the F_2_-*S*-DABO **38**, and the F_2_-*N*,*N*-DABO **116**) suppress HIV-1 replication for the entire experimental period (40 days) (virucidal action). After 4 h treatment, nevirapine and efavirenz are only virustatic, whereas **116** at 3.5 μM totally suppressed HIV-1 replication in cultures acutely infected with high multiplicity of infection (MoI = 5 CCID_50_/cell), allowing cell multiplication as in uninfected cultures for 40 days.^93^ In 2010/2011, **116** incorporated in a liposomal gel displayed protective properties against intravaginal challenge of Rhesus macaques with RT-SHIV, a chimeric simian immunodeficiency virus (SIV) containing the RT gene of HIV-1.^94,95^ Later (2013), the preclinical development of a **116**-releasing silicone elastomer vaginal ring (SEVR) was reported, including pharmacokinetic (PK) and efficacy studies in macaques.^96^ This medical device proved to partially protect macaques from the vaginal challenge, resulting in a practical method for providing sustained and coitally independent protection against vaginal exposure to HIV-1. In 2018, the Pedersen group explored the enantioselective synthesis of **116** and its analogues by asymmetric hydrogenation of 6-(1-arylvinyl)pyrimidine precursors of to the corresponding ethylidene derivatives using asymmetric rhodium(I), ruthenium(II), or iridium(I) catalysts.^97^ Although not decisive, this study was the first step in developing a true practical route for the scalable production of pure *(R)*-**116**, paving the way for future clinical use of the compound.

Other *NH*- and *N*,*N*-DABOs were reported in 2008 by Chen and co-workers, but with the 1-naphthylmethyl moiety at C6 and a set of *N*-substituents at C2 which looked very motley. The resulting compounds were much less potent than the 2,6-difluorobenzyl analogues, with EC_50_ values in the best cases just below 1 μM. Only data against HIV-1_IIIB_ and HIV-2_ROD_ were reported, with no activity against HIV-2; nothing was described about the effects against mutant strains as well as against both WT and mutant RTs.^98^

In 2011, a combination of the peculiar structural features from both DAPYs and DABOs led us to the development of a DAPY–DABO hybrid series, bearing (i) the 4-cyanoanilino moiety at C2 (typical of DAPYs) and (ii) the 2,6-difluorobenzyl or -(2-phenethyl) portion at C6 (typical of DABOs). The C4 position of the hybrids carried a hydroxy, chloro, amino, or hydrogen group. Such compounds showed a characteristic SAR profile and nanomolar anti-HIV-1 activity at both the enzymatic and cellular level. Among the tested compounds, **127**, **128**, and **129** (Figure 11) exhibited (sub)nanomolar activity against HIV-1 WT and clinically relevant mutant strains as well as against WT and mutated RTs.^99^

**Figure 12:**
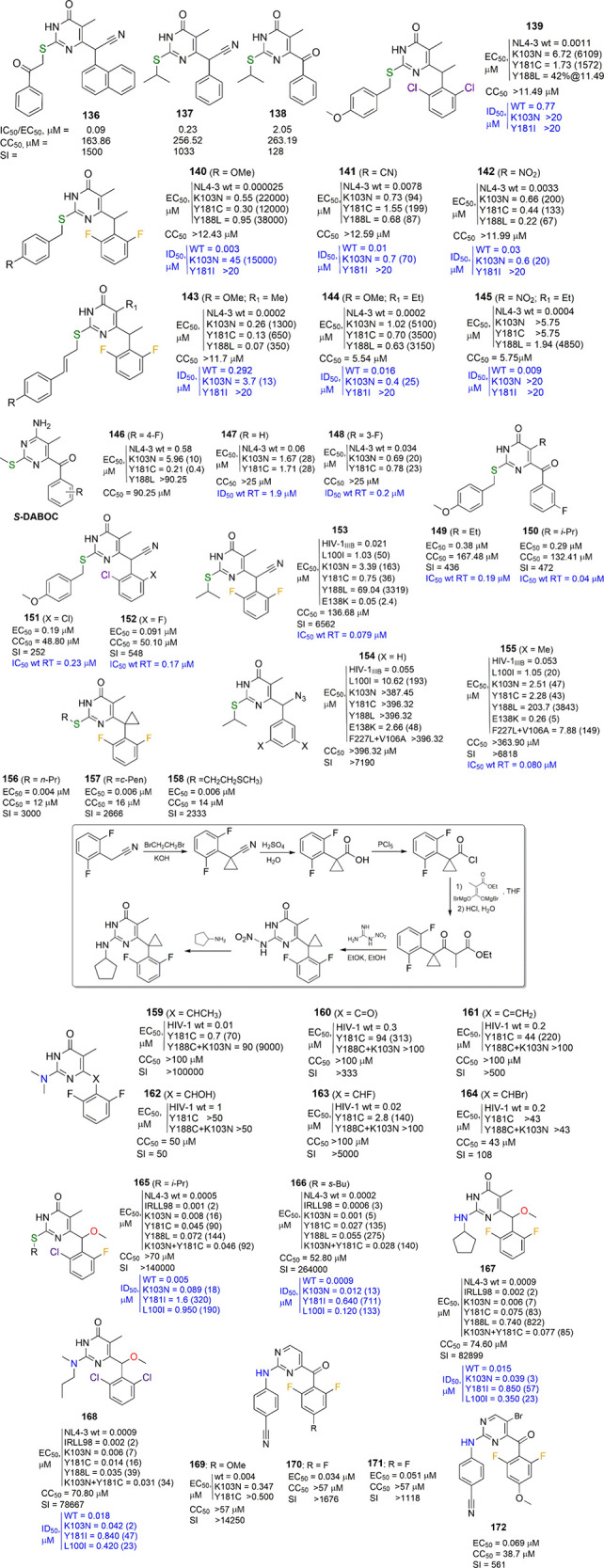
New *S*-, *NH*-, and *N*,*N*-DABO series and DAPY–DABO hybrids with changes at the C6 α-benzylic position and *S*-DABOC derivatives. EC_50_ (effective concentration able to induce 50% inhibition of HIV-1 cytopathic effect in MT-4 cells), CC_50_ (cytotoxic concentration able to reduce by 50% the viability of mock-infected cells), SI (selectivity index, ratio of CC_50_/EC_50_), and IC_50_/ID_50_ (concentration of compound able to inhibit by 50% the HIV-1 RT activity, in blue) are reported, when available. Fold-resistance is reported in brackets.

In 2012, Botta and his team reported novel *S*-DABO and *NH*/*N*,*N*-DABO analogues of their constrained hit compound, the 6-[1-(2,6-difluorophenyl)ethyl]-2-[(4-methoxybenzyl)thio]-5-methyl-4(3*H*)-pyrimidinone **140** (see below, Figure 12), bearing different linkers between the 4-methoxyphenyl fragment and the C2 position of the heterocyclic core.^100^ In particular, three-carbon atom chains with the insertion of double or triple bond, triazole ring, ketone, hydroxy, or oxime groups were used for *S*-DABOs. An *NH* or *N*-methyl group joined to an *N*-methylene or -cyclopropylmethyl portion was used for *NH*/*N*,*N*-DABOs. Surprisingly, all the *NH*/*N*,*N*-DABOs, while presenting the methyl substituent in the benzylic α-position, were prepared as 5-H and none as 5-methyl derivatives (**130**–**133**, Figure 11). Nevertheless, all of the *NH*/*N*,*N*-DABO compounds exhibited single-digit nanomolar potency against WT HIV-1 and retained some effectiveness against the K103N and Y181C mutant strains in infected cells, displaying similar behavior also in enzymatic assays. Unfortunately, they were highly cytotoxic or poorly soluble in water. Interestingly, this paper also reported the determination of the basic in vitro ADME properties of the title compounds and showed improved aqueous solubility for the *N*,*N*- and mainly the *NH*-DABOs with respect to the *S*-DABOs.^100^ The *N*,*N*-DABOs corresponding to the previous hit **132** but with a methyl group at C5 and alternatively a 1-(2,6-difluoro- or 2,6-dichlorophenyl)ethyl group at C6 (namely, **134** and **135**, Figure 11) were reported by the same group in 2016, in a subsequent study in which the new hit compound **134** was incorporated into a gel formulation and tested as a microbicide due to its very good profile toward HIV-1 mutant resistant strains.^101^ Unfortunately, no enzymatic data were reported for these last compounds.

## 10 DABOs with Changes at the α-Benzylic Position

In 2001, we had the intuition to insert a small alkyl group (methyl, ethyl) at the C6 α-benzylic position combined with a C5 alkyl substitution in the *S*-DABOs, with following conformational restriction of the whole structure and high improvement of the anti-HIV-1 inhibitory potency.^31^ Since 2007, different modifications at the *S*-DABO benzylic position have been carried out. An interesting work published by the Chen/De Clercq group was dedicated to the synthesis and SAR investigations of new (i) 6-[(α-cyano)benzyl] and (ii) 6-aroyl-substituted *S*-DABOs, the latter to be considered hybrids between *S*-DABOs and SJ-3366-type^75^ compounds.^102^ Upon biological screening on HIV-1-infected MT-4 cell cultures, most of the title compounds appeared to be endowed with moderate to low activity, with the best potency shown by (i) *S*-phenacyl or small *S*-alkyl groups at C2 and (ii) a phenyl or 1-naphthyl ring at C6 for the (α-cyano)benzyl derivatives and (i) an *S*-isopropyl or *S*-cyclopentyl group at C2 and (ii)a phenyl ring at C6 for the α-aroyl analogues (**136**–**138**, Figure 12).

Again in 2007, the Botta group published two articles on *S-*DABOs, exploring the effect of the insertion of a methyl or ethyl group at the α-benzylic position of their previously reported (phenylalkyl)-*S*-DABOs and replacing the α-benzylic position with a carbinol, carbonyl (this change is in analogy with Chen’s series), methyl-carbinol, and vinyl group. The first is a clear example of high-throughput screening of a library of compounds, obtained via a parallel solution-phase synthesis.^103^ Most of the prepared compounds, mainly due to the insertion of a small alkyl group at the methylene linker of the 2,6-dichloro- or 2,6-difluorobenzyl portion at the C6 position, showed pronounced anti-HIV-1 activity against the WT form of the virus but significantly lost potency against the mutant strains as well as against mutant RTs (**139**–**142**, Figure 12). The 6-[1-(2,6-difluorophenyl)ethyl]-2-(4-methoxybenzyl)thio-5-methyl-4-(3*H*)-pyrimidinone lead compound **140** was characterized by a very high potency against WT HIV-1 (EC_50_ = 25 pM) but showed several thousand fold resistance against the K103N, Y181C, or Y188L HIV-1 mutant strains in cells and against the K103N RT mutant form in the enzymatic assay. The two 4-CN- and 4-NO_2_-substituted benzylthio analogues **141** and **142** were less potent against WT HIV-1 (EC_50_ values = 7.8 and 3.3 nM, respectively) but exhibited much lower fold resistance against mutant strains and K103N RT (Figure 12). Similarly, the 2-(cinnamylthio) series described in the same paper^103^ included compounds with sub-nanomolar potency against WT HIV-1 and 350–5100-fold resistance against the tested mutant strains (**143**–**145**, Figure 12). Typically, all of these compounds were characterized by high cytotoxicity and/or low water solubility. The second paper was mostly dedicated to the synthesis of 6-benzoyl, 6-(1-hydroxy-1-phenylmethyl), 6-(1-hydroxy-1-phenylethyl), and 6-(1-phenylvinyl) pyrimidin-4(3*H*)-ones. No SAR of the aforementioned compounds was discussed, leaving the feeling that these chemical modifications led to very weak or inactive compounds.^104^ The 4-amino-6-(4-fluorobenzoyl)-5-methyl-2-methylthiopyrimidine **146** was the only analogue with reported, low antiviral activity (Figure 11).^104^ A continuation of this work showed that the removal of the 4-fluoro atom or better its replacement with a 3-fluoro substituent at the C6-benzoyl portion improved the anti-HIV-1 potency of these cytosine analogs, called *S*-DABOCs (**147** and **148**, Figure 12).^105^ Molecular modeling studies suggested important features responsible for the interaction of *S*-DABOCs and HIV-1 RT, interesting for further optimization of their structure. Further studies of the same research group led to the discovery of the 4-dialkylamino-6-vinylpyrimidines (DAVPs), whose peculiar features were their ability to compete with the natural nucleoside substrates of the HIV-1 RT and their ability to exert a noncovalent mechanism of action despite the presence of the vinyl group in their structure.^106−108^ However, regardless of the novelty and the unique biological properties of these compounds, their weak anti-HIV-1 activity seems to preclude their further development.

Another series of *S*-DABOC derivatives was described in 2010 by Qin et al.^109^ Only C6-benzyl-substituted compounds were synthesized, with (i) a (phenylalkyl)-/(2-naphthylmethyl)-thio chain at C2 and (ii) a 4-chloro or 4-amino substituent at the pyrimidine C4 position. All compounds were tested only against WT RT and not against mutant RTs or in HIV-1 infected cells, and all of them were practically inactive.

In 2013/2014, following their previous work,^102^ the group of Chen and co-workers described two series of 6-aroyl^110^ and 6-(α-cyanobenzyl)^111^ analogues bearing at the C2 position the same (4-methoxybenzyl)thio group used by the Botta group. Such compounds were endowed with, in the best cases, sub-micromolar potency against HIV-1-infected MT-4 cells (**149**–**152**, Figure 12). In the first series, the hit compounds were 3- and 22-fold less potent than nevirapine and efavirenz, used as reference drugs. In the latter series, the best compounds displayed the same potency as nevirapine and 10-fold lower potency than efavirenz. None of them retained antiviral activity against the HIV-1 K103N + Y181C double mutant strain or HIV-2. The chemical diversity of the α-benzylic cyano-substituted analogues was expanded in 2022 by the same research group with the insertion, at the C6 position of the 2-(isopropylthio)-5-methyl-4(3*H*)-pyrimidinone, a large number of variously substituted α-cyanobenzyl as well as α-cyano-2- or -3-pyridylmethyl groups, aiming at an interaction with the W229 within the RT catalytic pocket.^112^ Not surprisingly, the best compound was the one featuring the 6-(α-cyano-2,6-difluorobenzyl) group (**153**, Figure 12), which showed low nanomolar inhibition against WT HIV-1 and only 2.4-fold decrease in potency toward the E138K mutant. The molecule also exhibited favorable drug-like properties *in vitro* and *in vivo* without acute toxicity and optimal oral bioavailability in mouse models. One year later (2023), the same group described a series of C6 α-azidobenzyl-*S*-DABOs against WT HIV-1 and resistant mutants, but the best prototypes **154** and **155** did not surpass **153** in potency, likely because they contained a benzyl/3,5-dimethylbenzyl group instead of the 2,6-difluorobenzyl one.^113^

An original way to overcome the problem of enantioseparation of α-substituted *S*-DABOs (see below) was proposed in 2016 by Babushkin and coauthors,^114^ who reported a short series of 2-(alkylthio)-6-[1-(2,6-difluorophenyl)cyclopropyl]-5-methyl-4(3*H*)-pyrimidinone analogues (**156**–**158**, Figure 12) featuring the replacement of the C6 α-benzylic methyl/ethyl substituent with the achiral 1,1-cyclopropylidene group. In cellular assays, these compounds displayed up to single-digit nanomolar HIV-1 inhibition, being about 10-fold more potent than nevirapine and 2-fold less potent than efavirenz. The most potent compounds carried an *n*-propylthio, cyclopentylthio, or 2-(methylthio)ethyl substituent at C2. No data were reported about their activity against HIV-2 or HIV-1 mutant strains. Another 1,1-cyclopropylidene analog belonging to the F_2_-*NH*-DABO series, the 2-(cyclopentylamino)-6-[1-(2,6-difluorophenyl)cyclopropyl]-5-methyl-4(3*H*)-pyrimidinone, was prepared through a novel synthetic pathway involving the condensation of the appropriate β-oxoester with nitroguanidine and following displacement of the nitroamide from the obtained 2-*N*-nitroisocytosine with cyclopentylamine (Figure 12).^115^ Yet, no HIV-1 inhibitory data relative to this compound have been reported.

As mentioned above, our F_2_-*N*,*N*-DABO prototype **116** was taken as a model by other research groups to develop some related analogues. In 2010^89^ and in 2016,^90^ the Pedersen group reported a series of analogues of **116** with a carbonyl, vinyl, hydroxy, fluoro, or bromo group at the α-benzylic position (**159**–**164**, Figure 12), all being less potent than the parent compound against WT HIV-1 and the mutant Y181C and Y181C + K103N mutant strains in infected cells and, in a few cases, more cytotoxic. Other analogues of **116** showing sub-micromolar EC_50_ values (and similar cytotoxicity) against WT HIV-1 were the 6-(2-fluoro-6-methoxy)benzoyl derivative and the 6-(2,6-difluorobenzyl)-5-methyl-2-[*N*-methyl-*N*-(4-iodobenzyl)amino]-4-(3*H*)-pyrimidinone,^90^ the latter “inspired” by the (4-methoxybenzyl)thio group at C2 previously reported first by Botta and coauthors (in 2005)^66^ and then by us (in 2008).^77^ Anyway, such compounds also were 50- and 20-fold less potent than the reference compound **116**. No enzymatic data were reported for any of these derivatives.

The introduction of a methoxy group in the α-position of the C6 benzylic portion of *S*-, *NH*-, and *N*,*N*-DABOs was an intriguing alternative to the previously reported methyl substituent.^116^ Similar to methyl, the methoxy group can ensure a van der Waals interaction with the C5 methyl group with subsequent conformational constraint; on the other hand, it leads to the modification of the hydrophilic–lipophilic balance of the whole molecule. The most potent compounds (**165**–**168**, Figure 12) were active in the single digit nanomolar or picomolar range against HIV-1 WT, also retaining fair potency toward clinically relevant mutant strains, with EC_50_ values remaining in the low nanomolar range for most derivatives. Some 2,6-dihalobenzyl DAPY–DABO hybrids featuring the methoxy group at the α-benzylic position were synthesized by the reaction of *p*-cyanoanilines with 2-(nitroamino)-4(3*H*)-pyrimidinones in pivalic acid according to the procedure illustrated above. Unfortunately, no anti-HIV-1 activity data were reported for such compounds.^117,118^

DAPY–DABO hybrids were also designed and synthesized with a carbonyl group linking the pyrimidine and the phenyl ring at C6.^119^ The most potent compounds (**169**–**171**, Figure 12), bearing (i) a *p*-cyanoaniline moiety at C2, (ii) a 4-substituted 2,6-difluorobenzoyl group at C6, and (iii) no substituents at C4 and C5, displayed single- to double-digit nanomolar inhibition against WT HIV-1 in infected MT-4 cells and low cytotoxicity. The bromo atom was tolerated at C5 (**172**, Figure 12), but with an increase in cytotoxicity. The hit compound **169** exhibited sub-micromolar potency against the K103N mutant and no activity against the Y181C mutant in cellular assays, thus being much less potent than etravirine and efavirenz, used as reference drugs. No enzymatic data were reported in the study.

## 11 Chirality Issues in *S*-DABOs

In the F_2_-*S*-DABO series, one of the best compounds (**38**, Figure 5) was resolved in 2001 into two individual enantiomers by means of HPLC, and the (*R*)-enantiomer was found to be 350-times more potent than its optical antipode (Figure 5).^120^ In 2006, the analytical and semipreparative HPLC separation of further F_2_-*S*-DABOs was reported, characterizing the two forms of the *sec*-butylthio side chain at C2 as well as the two forms of the ethyl-substituted α-benzylic position at C6 by means of tandem normal phase HPLC and CD-analyses.^121^ A follow-up of this study was published in 2009 describing the synthesis, HPLC separation, and establishment of absolute configuration for new Cl_2_-*S-*DABOs with or without conformational restriction at the α-benzylic position.^122^ These works described a multidisciplinary approach based upon independent physical, synthetic, and chromatographic methods, indicating that the configuration of a chiral benzylic carbon atom in the new DABOs was responsible for the modulation of intensity and sign of the diagnostic Cotton effect around 245 nm. The structural data obtained during this work could be used for better understanding of the interactions of *S*-DABOs with their biological target during the rational structure-based design of their novel congeners.

**Figure 13:**
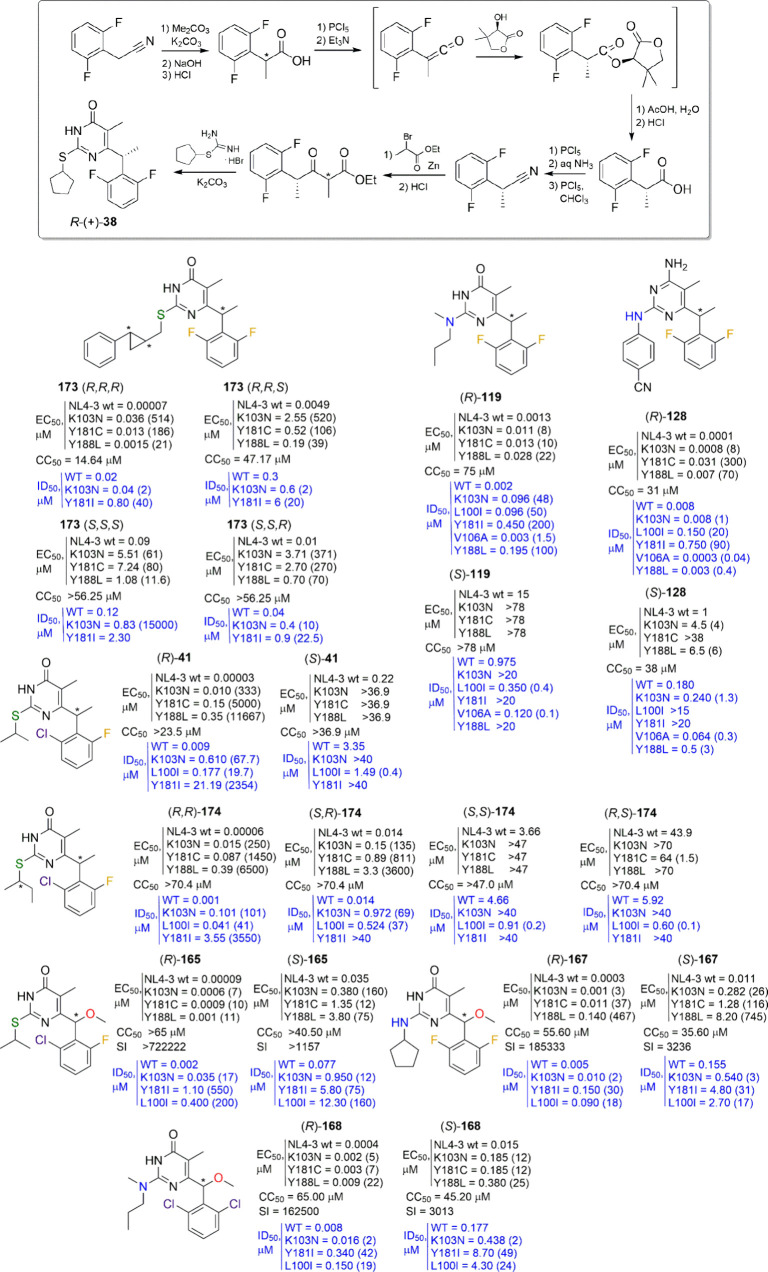
Enantioselective synthesis of *R*-(+)-**38**, C2-cyclopropylmethylthio-containing constrained F_2_-*S*-DABOs, and comparison between *R* and *S* enantiomers of the constrained DABOs **119**, **128**, **41**, and **174**, and the α-methoxy-DABOs **165**, **167**, and **168**. EC_50_ (effective concentration able to induce 50% inhibition of HIV-1 cytopathic effect in cells), CC_50_ (cytotoxic concentration able to reduce by 50% the viability of mock-infected cells), SI (selectivity index, ratio of CC_50_/EC_50_), and ID_50_ (concentration of compound able to inhibit by 50% the HIV-1 RT activity, blue) are reported in blue, when available. Fold-resistance is reported in brackets.

In the same year, we published the stereoselective synthesis of (*R*)-**38**, the 2-(cyclopentylthio)-6-[(1*R*)-1-(2,6-difluorophenyl)ethyl]-5-methyl-4(3*H*)-pyrimidinone,^123^ based on the known procedure for the preparation of individual enantiomers of 2-arylpropionic acids.^124^ The synthesis was achieved through the formation of a prochiral (2,6-difluorophenyl)methylketene which acylated the chiral d-(−)-pantolactone to furnish the optically active ester (3*R*)-4,4-dimethyl-2-oxotetrahydrofuran-3-yl (2*R*)-2-(2,6-difluorophenyl)propanoate. This intermediate was then subjected to acidic hydrolysis to (*R*)-2-(2,6-difluorophenyl)propanoic acid without isolation. Further conversion of this propanoic acid into the corresponding nitrile and its subsequent Blaise synthesis with ethyl 2-bromopropionate in the presence of zinc afforded the optically active 3-oxoester which gave (*R*)-**49** after condensation with *S*-cyclopentylisothiourea hydrobromide in basic medium (Figure 13).

In 2009, Botta and co-workers reported a novel series of structural analogues of their lead compound **140** (Figure 12), bearing a 1,2-cyclopropylidene linker between the 4-methoxyphenyl residue and the methylthio unit at the C2 position (compound **173**, Figure 13).^125^ The introduction of this type of linker into the structure of drug-like compounds is known to be somehow crucial for their activity, mainly due to the possibility of fixing the necessary conformation and reducing an entropy loss during the interaction with the biological target.^126^ In this way, it appeared possible to solve the problem of the loss of potency of the predecessor toward the clinically relevant HIV-1 strains, and particularly the K103N mutant. Indeed, compound **173** with the *R*,*R* cyclopropyl moiety on the left part of the molecule coupled with the (*R*)-α-methyl group at the benzyl at C6 was active at picomolar level against WT HIV-1 (similar to the 4-methoxybenzylthio analogue **140**, Figure 12) but, unlike **140**, retained nanomolar inhibitory potencies against the K103N, Y181C, and Y188L mutant strains. Again, the *R*,*R*,*R* configuration of the molecule was crucial for its inhibitory activity against both WT and mutant RTs (Figure 13) in enzymatic assays. Out of the two chiral parts of the molecule, the right one seems to modulate the activity against the Y181C and Y188L mutants, whereas the left seems to play a key role in the activity on the K103N mutant.

Further confirmation of the crucial role of the *R* configuration of the chiral center at the DABO α-benzylic position to obtain high anti-HIV-1 potency was provided by the enantioselectivity studies performed on the *N*,*N*-DABO **119** (Figure 11) and the DAPY–DABO hybrid **128** (Figure 11).^127^ The hybrid molecule **128** was approximately 4–44-fold more potent than (*R*)-**119** against WT HIV-1 and the K103N and Y188L mutant strains and practically equipotent toward the Y181C mutant strain. It is also remarkable that the loss of potency for the (*S*)*-*enantiomer of **119**, in comparison with the corresponding (*R*)-form, was much more evident than that for **128**. On the other hand, the latter compound proved to be ∼2-times more cytotoxic than **119** (Figure 13). Interestingly, (*R*)-**128** displayed faster binding to K103N RT with respect to WT RT, while (*R*)-**119** showed the opposite behavior.

In 2014, some representative 2-Cl-6-F-*S*-DABOs bearing one (**41**, see Figure 5) or two (**174**) stereogenic centers (Figure 13) were resolved into their stereoisomers and showed significant diastereo- and enantioselectivity in HIV-1 inhibition, confirming the correlation of the most potent antiviral activity with the *R* absolute configuration at the C6 α-benzylic position. In **174**, with two stereo centers, the C6 α-benzylic substituent drove the highest potency with the (*R*) configuration, and the C2 (*sec*-butyl)thio group furnished at least 10-fold increased potency with the *R* configuration, as well.^35^ Some years later, HPLC enantioseparation of three of the most potent *S*-, *NH*-, and *N*,*N*-DABOs carrying the methoxy substituent at the C6 α-benzylic position (**165**, **167**, and **168**, respectively, see Figure 12) yielded individual enantiomers of which again the *R* forms were the most potent for HIV-1 inhibition with picomolar inhibition of WT HIV-1 and low nanomolar inhibition of the tested mutant strains (Figure 13).^116^

## 12 DABOs with Alicyclic Structures at the C6-Methylene Position

In the late 1990s, the HEPT derivative emivirine was optimized by replacing the benzyl group at the C6 position of the uracil ring with a cyclohexylmethyl moiety, obtaining TNK-6123, which showed ∼30-fold greater inhibitory potency than emivirine against the clinically important Y181C and K103N mutant virus strains.^42^ In 2003, the Pedersen group reported a few 1*H*-imidazoles as “decarbonylated” DABOs, which when bearing a cyclohexylmethyl moiety at the imidazole C5 position displayed sub-micromolar inhibitory activity against HIV-1 (see **49**–**51**, Figure 6).^41^

**Figure 14:**
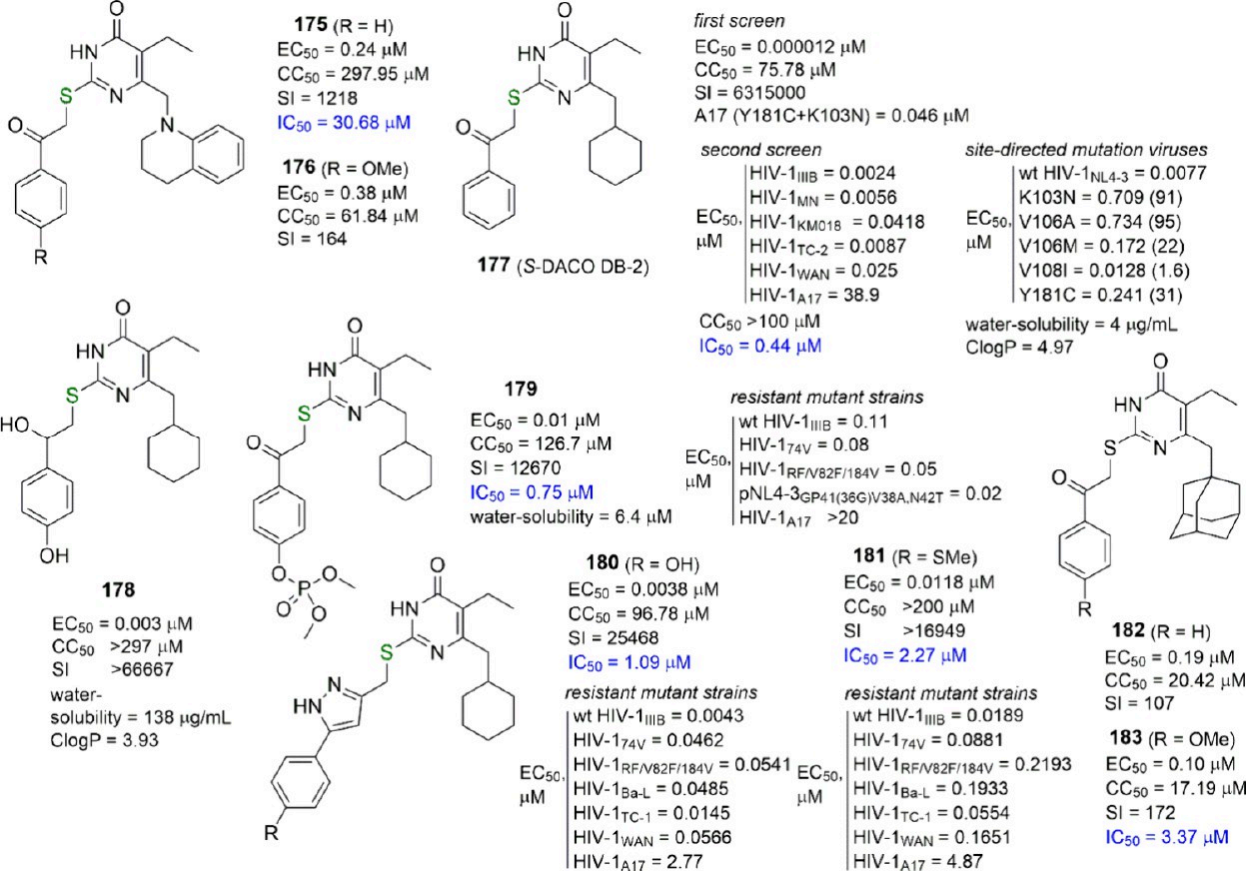
4-(3*H*)-Pyrimidinones carrying alicyclic substituents at the C6-methylene position. EC_50_ (effective concentration able to induce 50% inhibition of HIV-1 cytopathic effect in cells), CC_50_ (cytotoxic concentration able to reduce by 50% the viability of mock-infected cells), SI (selectivity index, ratio of CC_50_/EC_50_), and IC_50_ (concentration of compound able to inhibit by 50% the HIV-1 RT activity, in blue) are reported when available. Fold-resistance is reported in brackets.

In 2011, Liu and co-workers explored the effect of the introduction of a 1,2,3,4-tetrahydroquinolin-1-ylmethyl moiety at C6.^128^ The hit compounds (**175** and **176**, Figure 14) bearing an ethyl group at C5 and a 4-substituted (phenylacetyl)thio moiety at C2, showed sub-micromolar potency in WT HIV-1-infected cells that did not exceed those of nevirapine and delavirdine and was much lower than that of efavirenz, used as referenced drugs. None of the tested compounds showed any activity against the HIV-2 ROD strain. Prototype **175** exhibited high micromolar activity against WT HIV-1 RT. No data were reported about the effect on mutant strains in cells or mutant RTs in enzymatic assays.

In the same year, interesting results were obtained by He and co-workers, who introduced a cyclohexylmethyl at C6 in the *S*-DABO structure (*S*-DACOs).^44^ The lead compounds of this series carried a phenacylthio group at C2 and an ethyl or isopropyl substituent at C5 [**177** (DB-2), Figure 14], and displayed picomolar EC_50_ values toward WT HIV-1 in C8166 cells and unprecedented SIs. Remarkably, the structural analogues with the benzyl group replacing the cyclohexylmethyl at C6 showed 560–6667-fold lower potency against WT HIV-1, as well as the derivatives containing an isobutylthio group instead of the phenacylthio moiety at C2 (3720–8417-fold lower potency). Further studies performed on **177** in 2013 by the same research team reported a 200-fold decrease in the anti-HIV-1 activity, with the EC_50_ value increasing from 12 pM to 2.4 nM. The EC_50_ values against the clinical isolates KM018, TC-2, and WAN were in the 8.74–41.8 nM range, whereas different from what was reported in the previous paper the EC_50_ of **177** against the A17 strain carrying the Y181C + K103N mutation was ∼40 μM, thus showing the insurgence of resistance. Compound **177** showed low to moderate resistance values (1.6–91-fold) against site-directed mutated viruses, in comparison with the WT HIV-1_NL4–3_ strain. With respect to other *S*-DABOs, **177** displayed an improved sensitivity against K103N or Y181C mutants.^47^ Because of its high molecular hydrophobicity, **177** displayed a poor aqueous solubility and oral bioavailability. Therefore, the same authors prepared an analogue carrying a 4-hydroxy substitution at the phenacylthio portion at C2 and showing the ketone group reduced to the corresponding alcohol.^46^ This compound (**178**, Figure 14) showed similar anti-HIV-1 activity as **177** but improved water solubility and reduced ClogP. In 2023, another strategy adopted by the same group to overcome the low water solubility of **177** was the development of its phosphate esters at the C2 (4-hydroxyphenacyl)thio chain. In particular, the dimethyl ester (**179**, Figure 14) with respect to **177** was 20-fold more potent against WT HIV-1, from 2- to 10-fold more potent against HIV-1 mutant strains, and 17-fold more water-soluble. Unfortunately, it was also 4.5 times more cytotoxic. Moreover, in a pharmacokinetic test in rats, **179** demonstrated significantly improved oral bioavailability of 14.6%.^45^ Of note, in this work the potency of **177** dropped further: the EC_50_ vs WT HIV-1 here was 0.2 μM.

Replacement of the phenylacetylthio chain at C2 with a [(5-aryl-1*H*-pyrazol-3-yl)methyl]thio group furnished new highly potent *S*-DACOs with single-digit nanomolar effect on WT HIV-1 and slightly lower potency against resistant mutant strains, together with low cytotoxicity as well as acceptable pharmacokinetic properties and bioavailability in rats (**180** and **181**, Figure 14).^129^ The same phenacylthio and [(5-aryl-1*H*-pyrazol-3-yl)methyl]thio chains at C2 were inserted in analogues in which the cyclohexylmethyl at C6 was replaced by a (1,3-benzodioxazol-5-yl)methyl group, but in this case the potency against WT HIV-1 was only at low micromolar/sub-micromolar level,^130^ thus confirming the crucial role of the more flexible cyclohexylmethyl group to ensure better fit at the inhibition site, higher potency, and good maintenance of inhibition against mutant strains.

Anyway, the anti-HIV-1 potency decreased with the increase of the size of the cyclohexyl moiety at C6: indeed, in 2016, Liu et al.^132^ reported a series of 6-(1-adamantylmethyl)-substituted 4(3*H*)-pyrimidinones endowed with moderate activity against WT HIV-1 and EC_50_ values ranging from 0.10 to 5.39 μM. The 1-adamantylmethyl group has been known for 10 years as a substituent at the C6 position of 2-thiouracils and isocytosines,^131^ even if without data on antiviral activity. The most potent compounds in the above series carried an ethyl group at C5 and a substituted phenacylthio or (*N*-phenylacetamido)thio portion at C2 (**182** and **183**, Figure 14),^132^ but their high sub-micromolar EC_50_ values, together with the moderate cytotoxicity, clearly demonstrated that a rigid adamantane cage at C6 is too big to be an adequate replacement for the benzene or cyclohexane ring.

## 13 Molecular Modeling Techniques Used for the Design of DABO and Related Derivatives

The development of effective HIV-1 NNRTIs has involved various computational and experimental techniques. These techniques included Comparative Binding Energy (COMBINE) modeling,^133^ three-dimensional quantitative structure–activity relationships (3D-QSAR) such as comparative molecular field analysis (CoMFA)^134^ and comparative molecular similarity analysis (CoMSIA),^135^ molecular docking,^136^ molecular dynamics (MD),^137^ and application of machine learning algorithms.^138^ Collectively, these techniques provided insights into structural modifications that led to improved binding, potency, and selectivity of DABOs and their derivatives against both wild-type and drug-resistant HIV-1 RT.

### 13.1 COMBINE Modeling

The COMBINE method, and specifically the enhanced COMBINEr approach, was employed to create a structure-based 3D-QSAR model of HIV-1 RT, covering both wild-type and various drug-resistant mutants. COMBINE focuses on ligand and receptor interactions by analyzing specific energetic contributions, such as electrostatic, steric, and desolvation energies, at the amino acid level within the RT enzyme. The COMBINEr variant used here integrates both ligand- and structure-based alignments for accuracy. The study examined seven RT enzyme variants in complexes with nevirapine and efavirenz, resulting in a comprehensive training set of 14 complexes. The COMBINEr model yielded a predictive accuracy with an average error of 0.89 p*K*_i_ units, successfully capturing both wild-type and mutant RT potency. This model also elucidated the importance of steric and hydrophobic contacts within the NNRTI binding pocket (NNBP) for stabilizing DABO analogs.^81,139^ COMBINEr analysis highlighted the importance of steric and hydrophobic interactions in the NNBP, especially around residues such as Leu100, Lys103, and Tyr181 and their mutant forms (L100I, K103N, and Y181C). This provided insights into how DABO modifications could stabilize interactions even when facing mutations that alter the NNBP conformational shape. A subsequent application of the COMBINEr model was used to inspect and indicate the binding mode of a chiral amino-DABO with very good precision.^127^ In a similar COMBINEr approach, enantioselective binding studies on the racemic F_2_-*S*-DABO **38** revealed that the *R* enantiomer demonstrated higher activity due to its enhanced binding affinity within the RT non-nucleoside binding pocket (NNBP). This chiral specificity underscores the significance of stereochemistry in NNRTI design and suggests the potential of enantiomerically pure compounds for increased efficacy against HIV.^120^

### 13.2 3D-QSAR

As reported in a variety of papers, 3D-QSAR methods provided insights into the steric and electrostatic influences on the potency of DABO analogues. CoMFA and CoMSIA achieved cross-validated correlation coefficient *r*^2^ (*q*^2^) values of 0.636 and 0.655, respectively, with external predictive coefficient *r*_pred_^2^ values of 0.907 (CoMFA) and 0.886 (CoMSIA). An in-depth analysis of the models revealed some interesting key features:

- Steric bulk at C6 and electron-rich groups at C2 and C5 significantly increase DABO binding affinity.
- Bulky groups in C6-modified *S*-DABOs, such as the 3,5-difluorobenzoyl analogue of **139** (see Figure 11), demonstrated high antiviral efficacy (EC_50_ of 0.26 μM and SI of 541) by promoting additional hydrophobic contacts with NNBP residues.^110^

In the case of *S*-DABOC analogs, modifications at the C6 position (e.g., substitution of benzyl with fluorinated aryl groups) improved resistance profiles against common mutations such as Y181C and K103N. CoMFA/CoMSIA contour maps highlighted that electron-donating substituents at C6 favor binding within the hydrophobic NNBP, especially among *S*-DABOC derivatives with improved activity against mutant RT.^105,106^

### 13.3 Molecular Docking

Docking simulations across various DABO derivatives revealed interactions critical for NNRTI efficacy, particularly those with Lys101, Tyr181, and Trp229. These residues support the hydrophobic and hydrogen bonding interactions that are essential for stabilizing DABOs within the NNBP of the RT enzyme.^140^ From molecular docking investigations, the following insights were drawn:

- Hydrophobic residues surrounding the C6 position create a favorable binding environment for bulky, fluorinated aryl groups. A compound similar to **105**–**108** (see Figure 9), bearing a 2-chloro-6-fluorobenzyl at C6, an ethyl group at C5, and a (phenylaminocarbonyl)methylthio chain at C2, achieved EC_50_ values of 0.19 μM against wild-type RT and showed efficacy against resistant mutants such as E138K and Y181C (EC_50_ values = 2.03 and 2.13 μM, respectively).^110,80^
- In *S*-DABOC analogs, the cytosine-modified structure achieved selective inhibition by interacting with the RT NNBP without relying on residues commonly associated with resistance mutations, such as Y181 and K103.^105,106^

Molecular docking confirmed that interactions with Lys101 and Tyr318 were critical to RT inhibition, particularly for analogs with amino- or electron-rich groups at C2. Compound **114** (Figure 11) showed promising results, achieving nanomolar IC_50_ values against wild-type and mutant RT forms, suggesting its potential as a competitive alternative to efavirenz.^81^

### 13.4 Molecular Dynamics

MD simulations complemented the docking studies by capturing the dynamic stability of the DABO–RT interactions. These simulations highlighted the role of hydrophobicity around the C6 position and the contribution of flexible linkers in maintaining stable interactions under varying conformational states of the RT enzyme. From MD runs the following was observed:

- Hydrophobic residues, including Trp229 and Tyr318, support the stability of bulky C6-substituted DABOs during RT binding, which is in good agreement with SAR findings favoring bulky substituents for improved potency.^141,142^
- The simulations suggested that hydrophobic and π-stacking interactions contribute to DABO stabilization, especially when bulky substituents reduce spatial fluctuations in the NNBP.

### 13.5 Machine Learning

Using Decision Tree and Random Forest algorithms and molecular descriptors like topological indices and lipophilicity classification models were derived on a DABO analog series. The Random Forest model achieved 85% classification accuracy, while the Decision Tree model had 98% accuracy on training data (77% cross-validation accuracy). Key descriptors linked activity to high lipophilicity and structural complexity, suggesting that hydrophobic and topologically connected structures improve RT binding affinity.^143,144^ Support Vector Machine (SVM), neural networks, and multiple linear regression (MLR) were used to predict binding affinities based on molecular descriptors. SVM outperformed other models, showing *r*^2^ = 0.939 and *q*^2^ = 0.876, suggesting a nonlinear relationship between hydrophobicity, electronic features, and DABO activity. Key descriptors included C6 substituent hydrophobicity and aromaticity, which are critical for maintaining high activity across resistant strains. The successful predictions by SVM provided criteria for designing new DABO derivatives with enhanced potency.^80,142^

### 13.6 De Novo Virtual Screening

Virtual screening and molecular modeling efforts focused on optimizing DABO analogs for potency against mutated RTs. This led to the identification of promising substitutions, particularly trisubstituted aromatic rings at C6, that maximize binding efficiency without steric clashes in the NNBP. In silico design thus offers a cost-effective way to identify new DABO derivatives with potential clinical relevance.^105,142^

Thus, the integration of CoMFA, CoMSIA, COMBINEr, docking, MD, and machine learning could offer a comprehensive approach to designing new DABO-based NNRTIs with improved efficacy and selectivity.

## 14 Binding Mode of DABO Derivatives

As stated above, it was very difficult to obtain high-resolution X-ray structures with DABOs, the only isolated crystals being published in 1996, 1998, and 2006^16−18^ with weak crystal structures, due to huge difficulty in purification. For this reason, we and others have never succeeded in gaining such structural biology information on DABOs in complex with HIV-1 RT but have performed molecular modeling studies to highlight the possible binding mode of these compounds.

As is known, the mechanism of action of DABOs and their congeners involves their binding to the NNBP of RT, an allosteric hydrophobic cavity located within the p66 subunit, approximately 10 Å away from the polymerase active site. The NNBP, absent in the NNRTI unliganded form of RT, is opened during RT binding to the nucleic acid (RNA or DNA) and optionally also in the presence of a three-phosphatized nucleoside resulting in the rearrangement of key residues, including Lys101, Lys103, Val179, Trp229 Tyr181, Tyr188, Phe227, Trp229, and Tyr318, forming a hydrophobic pocket with hydrogen bonding anchoring sites to which the NNRTIs bind. The formation of RT/NNRTI in this structural rearrangement is critical, as it locks the enzyme in an open conformation, restricting the thumb domain’s flexibility and thereby preventing efficient RNA/DNA synthesis.^105,141^ Molecular dynamics simulations have confirmed that the hydrophobic interactions with residues such as Val179, Phe227, and Trp229 stabilize the ligand–enzyme complex, while π–π stacking interactions between the aromatic moieties of the inhibitors and residues like Trp188 and Phe227 further enhance the binding affinity.^106,127^

In addition to hydrophobic interactions, hydrogen bonding plays a pivotal role in determining the binding efficiency of the DABOs. In the NNBP closed form, an important electrostatic interaction is established between Lys103 and Tyr181; therefore, key hydrogen bonds are often observed between the ligand and residues such as Lys101, Lys103, and Tyr318, contributing to the orientation and stabilization of the inhibitor within the NNBP.

Either hydrophobic or hydrogen bonding interactions are particularly significant for inhibitors targeting drug-resistant RT mutants; in particular, compensatory hydrogen bonding helps in maintaining the binding capacity to the hydrophobic mutated RTs. For example, the introduction of an amide fragment in the C2-side chain of *S*-DABOs^80^ or the sulfur-to-amino group substitution at the DABO C2 position^81^ introduced a new anchor point, facilitating hydrogen bond formation with Lys101 and counteracting resistance mutations such as Y181C and K103N.

Molecular docking proved to be an effective tool to investigate the binding mode of NNRTIs^136^ and has further demonstrated that DABOs adopt a butterfly-like conformation within the NNBP, which is essential for optimizing their steric and electronic interactions with the enzyme.^142,145^

To improve the efficacy of DABOs, systematic modifications at critical positions of the pyrimidine scaffold have been explored. Bulky or hydrophobic groups inserted at the C5 and C6 positions exploited the NNBP hydrophobic environment, leading to improved binding affinity and selectivity. Furthermore, the introduction of polar or aromatic substituents at the C2 position strengthened both hydrophobic and hydrogen-bonding interactions, revealed to be critical for countering resistance mutations.^81,105,106^

**Figure 15:**
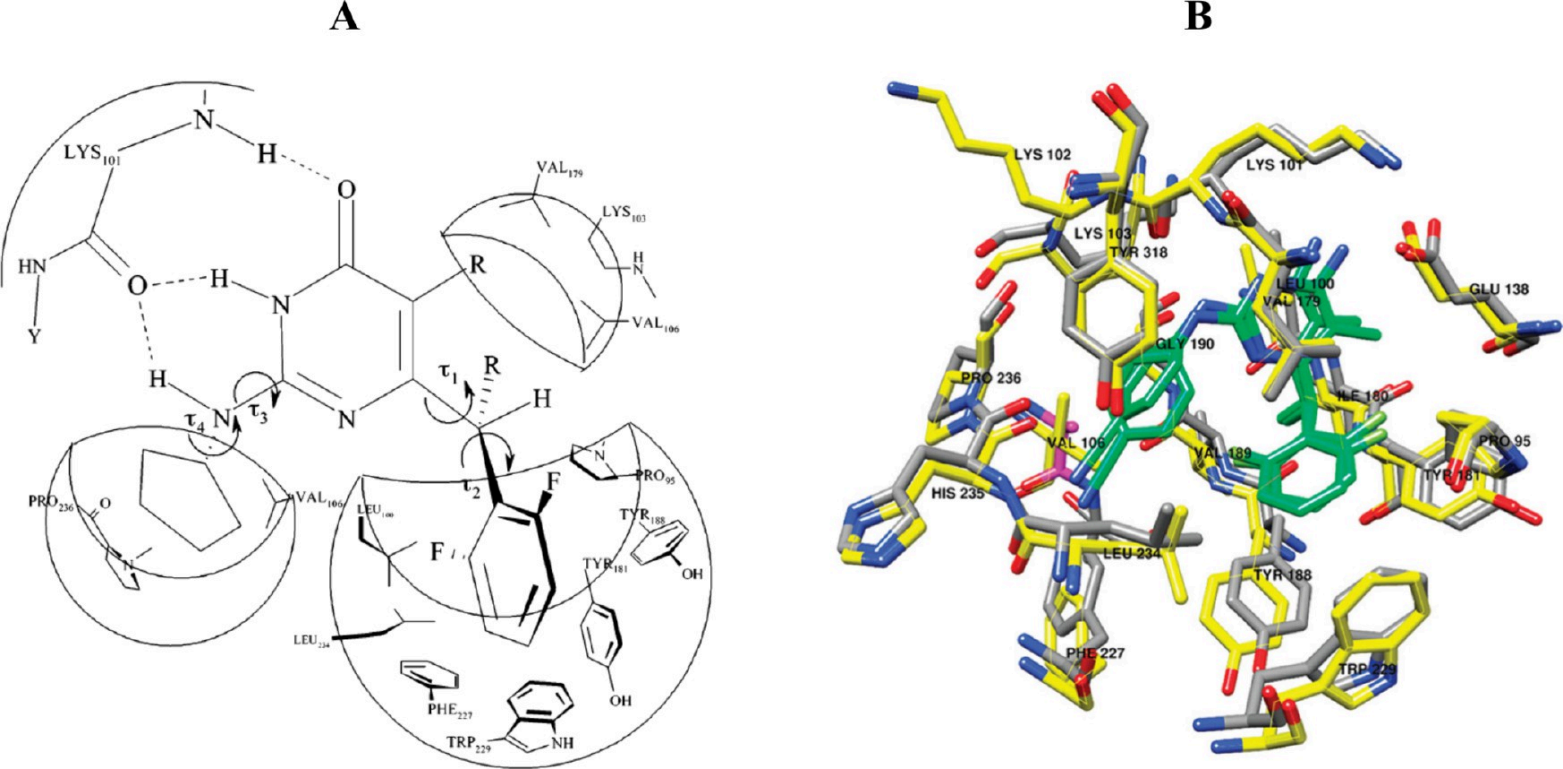
(A) Scheme of the F_2_-*NH*-DABOs/RT binding mode interactions. Reproduced from ref (81). Copyright 2004 American Chemical Society. (B) Comparison between (*R*)-**128** (green carbon atoms) docked into WT (yellow) and V106A mutated (gray) RT. The only residue that appreciably moves is Tyr188. The mutated Ala106 is displayed in magenta. Reproduced from ref (127). Copyright 2012 American Chemical Society.

Notably, a multidisciplinary approach including synthesis, molecular docking, and COMBINE revealed that insertion of a chiral center at the C6 carbon atom led to (*R*)-enantiomers of DABOs often displaying superior inhibitory activity compared to their (*S*)-counterparts, attributed to better spatial alignment within the NNBP and enhanced interaction with key residues^80,106,127^ (Figure 15).

Furthermore, application of several computational tools, including 3D-QSAR modeling using CoMFA, CoMSIA, and COMBINE, provided deeper insights into the structural determinants of DABO activity. These models highlight the importance of steric, hydrophobic, and electrostatic fields in dictating the inhibitory potency. Validation of these models through test sets has demonstrated their robustness and predictive power, guiding the rational design of second-generation NNRTIs.^141,142,145^

Moreover, molecular dynamics simulations have been instrumental in elucidating the dynamic stability of the ligand–RT complexes, providing valuable insights into how specific modifications impact binding affinity and resistance profiles.^80,106^

The integration of experimental with computational approaches, such as molecular modeling, docking, and structure-based drug design, enabled the detailed characterization of DABOs as potent NNRTIs. These studies have not only elucidated the molecular basis of their interaction with HIV-1 RT but also aided the design of inhibitors with enhanced efficacy against both wild-type and drug-resistant viral strains.^105,127,142,145^

## 15 Non-Anti-HIV-1 RT Uses of DABOs

**Figure 16:**
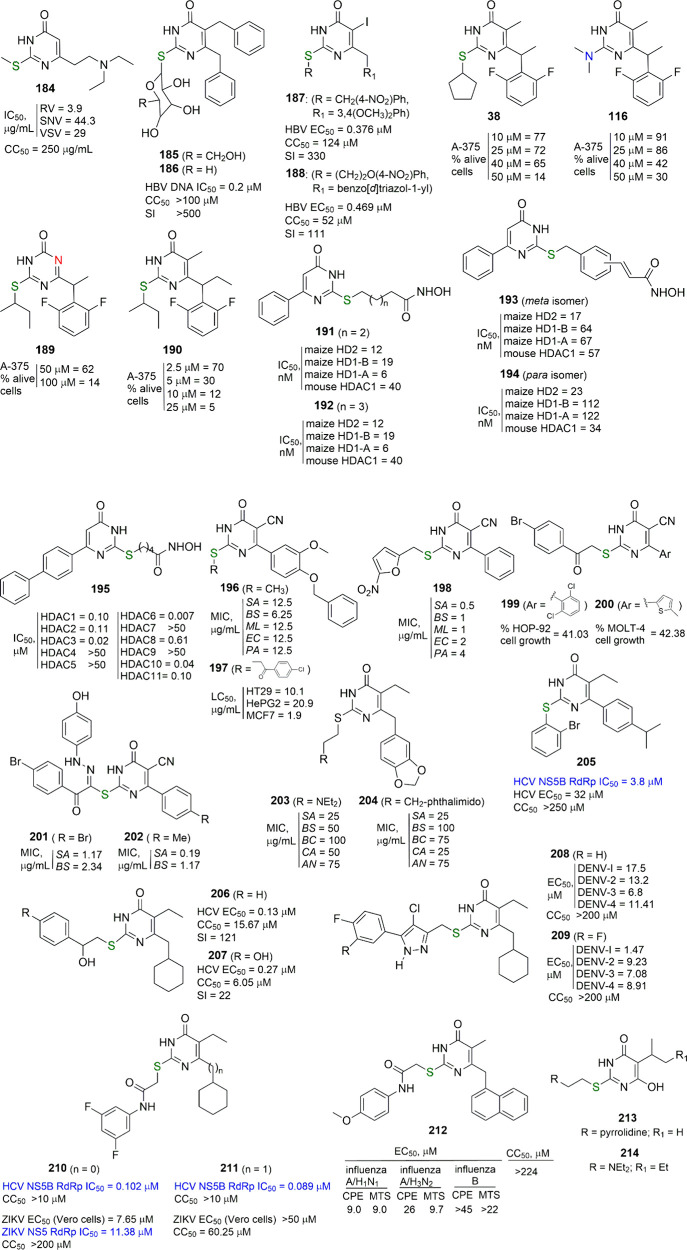
4(3*H*)-Pyrimidinones with activities other than HIV-1 RT inhibition. RV, rubella virus; SV, Sindbis virus; VSV, vesicular stomatitis virus. *SA*, *S. aureus*; *BS*, *B. subtilis*; *ML*, *M. luteus*; *EC*, *Escherichia coli*; *PA*, *P. aeruginosa*; *BC*, *Bacillus cereus*; *CA*, *Candida albicans*; *AN*, *Aspergillus niger*. RdRp, RNA-dependent RNA polymerase; CPE, cytopathic effect; MTS, formazan-based MTS assay. Enzyme data are reported in blue when available.

Since the late 1990s, some papers describing alternative DABO biological activities have been published. The Botta group in 1999 and 2002 reported that some 4(3*H*)-pyrimidinone analogues bearing an aminoalkyl group at C6 such as **184** (Figure 16), with very low (if any) activity against HIV-1, showed encouraging inhibition against Sindbis, vesicular stomatitis, and mainly rubella viruses.^146,147^

A paper by Aal in 2002 described a series of novel *S*-DABO analogues carrying a 3,4-dimethoxybenzyl group at C6 as novel effective HBV replication inhibitors.^148^ Unfortunately, no numerical value of the potency or cytotoxicity of the title compounds was reported. In a sequel (2010) of his previous work, the same author reported some 5,6-dibenzyl-2-thiouracils containing a carbohydrate residue linked to the C2 sulfur atom, a peculiar feature which had never been used previously in *S*-DABOs, as HBV-replication inhibitors.^149^ The most potent compounds, bearing β-d-galactopyranosyl- or β-glucopyranosyl substituents (**185** and **186**, Figure 16), showed IC_50_ values of 0.2 μM in HBV-infected cells, being 2 times less potent than the reference lamivudine.

Another study, inspired by the work of Aal and reported by Liu, Wang and co-workers in 2015, described some 5-iodo-2-(4-substituted benzyl)thio- or -(2-phenoxyethyl)thio-4(3*H*)-pyrimidinones with anti-HBV activity.^150^ The most potent compounds had a 3,4-dimethoxybenzyl or (benzo[*d*]triazol-1-yl)methyl portion at C6 and were 2-fold more potent than lamivudine (**187** and **188**, Figure 16).

In 2005 two F_2_-DABO prototypes, **38** and **116**, were tested against human endogenous non-telomeric RT (endo-RT) in human differentiating cell systems to investigate their antiproliferative and cytodifferentiating activity in the A-375 human melanoma cells. These compounds significantly reduced cell proliferation with only a low induction of cell death accounting for apoptosis and without significant nonspecific toxicity, thus suggesting that the observed reduced cell proliferation was mainly due to cell cycle arrest or delay. Moreover, the two compounds facilitated the morphological differentiation of cells, showing action at the epigenetic level in cell transformation and tumor progression (Figure 16).^151^ These results prompted the synthesis of some related 1,3,5-triazine-2(1*H*)-ones (ADATs), carrying a new heterocyclic core.^152^ Among the tested compounds, 6-(*sec*-butylthio)-4-[1-(2,6-difluorophenyl)ethyl]-1,3,5-triazine-2(1*H*)-one **189** (Figure 16) appeared to be the most effective antiproliferative agent. At the same time, the corresponding 6-[1-(2,6-difluorophenyl)propyl]-2-thio-5-azauracil, although not affecting cell proliferation, showed a strong cytodifferentiation effect that may be caused by a marked up-regulation of the *e-cad* gene. The F_2_-*S*-DABO analogue **190**, synthesized later (2011), proved to have stronger antiproliferative and cytodifferentiation effects than the other tested derivatives in A375 melanoma cells (Figure 16), with induction of apoptosis in a cell-density-dependent manner and antagonism of tumor growth in animal models.^153^ As expected, one of the isomers with the *R* configuration at the ethyl-substituted α-benzylic position was the most potent among the four possible stereoisomers. Treatment of PC3 metastatic prostate carcinoma cells with this isomer of **190** resulted in decreased proliferation and, at the same time, induced genomic damage associated with rearrangements of the nuclear architecture, particularly at peripheral chromatin, disruption of the nuclear lamina, and budding of micronuclei. Such changes were reversible upon discontinuation of the RT inhibitory treatment, with reconstitution of the lamina and resumption of the cancer cell original features. Autophagy was responsible for the antiproliferative effect of **190**, as proved by the use of pharmacological autophagy inhibitors. Noteworthy, these alterations were not induced in non-cancer cell lines exposed to RT inhibitors.^154^ Moreover, **190** in combination with MAPK inhibitors strongly inhibited BRAF mutant melanoma cell growth, inducing apoptosis and delaying the emergence of resistance to targeted therapy.^155^

Starting in 2005–2006, we reported a series of 4-(3*H*)-pyrimidinones substituted with either a thio-polymethylene-hydroxamate or thiomethyl-cinnamyl-hydroxamate group at C2 and a phenyl or phenylalkyl group at C6.^156,157^ Such compounds (**191**–**195**, Figure 16) displayed histone deacetylase (HDAC) inhibition in the nanomolar range, similar to the reference suberoylanilide hydroxamic acid (SAHA), which is a well-known HDAC inhibitor. Indeed, with this structure they follow the described pharmacophore model for HDAC inhibition, which is made up of a cap group (here the C6 substituent) joined to a polar connection unit [the 2-mercapto-4(3*H*)-pyrimidinone fragment] and connected to a terminal zinc-binding group (the hydroxamate group) through a hydrophobic spacer (the polymethylene or cinnamyl portion).^158^ Replacement of the hydroxamate group with a 2′-aminoanilide or 2′-aminoanilide-like group retained the anti-HDAC effect of the compounds.^159^ When tested in cancer cells including cancer stem cells, these uracil-based hydroxamic acids (UBHAs) showed antiproliferative and cytodifferentiation effects, joined to increased histone and (in some cases) α-tubulin acetylation and p21 induction.^160,161^ Some chloro-substituted analogues remarkably potentiated the antifungal effect of fluconazole with respect to *Candida albicans* growth and were able to inhibit the fluconazole-induced resistance induction in *Candida* cultures.^162^ In addition, the hit compound **195** exhibited the ability to reactivate HIV-1 from latency,^163^ as well as antimalarial and anti-*Toxoplasma gondii* effects *in vitro*.^164−166^ Unfortunately, it failed when tested in an *in vivo* malaria mouse model.^164^

A little later, a series of *S-*DABO-like 6-[4-(benzyloxy)-3-methoxyphenyl]-5-cyano-2-alkylthio-4(3*H*)-pyrimidinones were screened for antimicrobial and antineoplastic activity by Rostom and co-workers.^167^ Among the tested compounds, the 2-methylthio analogue **196** exhibited the highest antibacterial effect showing twice the potency of ampicillin against *Bacillus subtilis* and the same potency of ampicillin against *Micrococcus luteus* and *Pseudomonas aeruginosa*, together with moderate antifungal activity (Figure 16). On the other hand, its *S*-(4-chlorophenacyl)-substituted analogue **197** showed the most pronounced activity against colon carcinoma HT-29 and breast cancer MCF7 human cell lines (Figure 16). Further development of such compounds led to **198**, carrying a 2-[(5-nitrofuran-2-yl)methyl]thio portion at C2 and endowed with higher potency than ampicillin against *Staphylococcus aureus* and *M. luteus* (Figure 16).^168^

As a follow-up of these studies, in 2012 Abou-Seri et al. reported some 5-cyano-6-aryl-4(3*H*)-pyrimidinones structurally related to *S*-DABOs and endowed with antimicrobial and anticancer activities.^169^ Among the described compounds, **199** and **200**, bearing a 2,6-dichlorophenyl or a 5-methyl-2-thienyl substitution at C6, respectively, and a (4-bromophenacyl)thio group at C2, showed a pronounced inhibitory effect (>50% at 10 μM) toward non-small-cell lung cancer HOP-92 and leukemia MOLT-4 cell lines, respectively, while **201** and **202**, bearing a 4-bromo- or 4-methylphenyl group at C6, respectively, and a (4-hydroxyphenyl)hydrazono moiety inserted on the (4-bromophenacyl)thio substituent at C2, exhibited the highest activity against *S. aureus* and *B. subtilis*, equal to or higher than that of amoxicillin, used as a reference drug (Figure 16). Regarding the molecular mechanism of the antimicrobial action of these compounds, the SecA ATPase and protein translocase, a critical member of the Sec family important in the translocation of membrane and secreted polypeptides/proteins in bacteria and essential for bacterial survival, may be involved.^170^

Antimicrobial and antifungal evaluations were also performed on a series of *S*-DABOs substituted at the C2 position with an (aminoalkyl)thio or a phthalimidoalkyl chain, which were reported two years later in three papers by El-Brollosy and co-workers (**203** and **204**, Figure 16).^171−173^ The synthesized compounds showed weak activity in both assays, suggesting that further structural optimization is needed to obtain more potent analogues.

Some DABO-like compounds were reported as anti-hepatitis C virus (anti-HCV) agents. A series of 5-cyano-6-phenyl-2-benzylthio-4(3*H*)-pyrimidinones like those reported as antimicrobial and anticancer compounds was described by Ding and co-workers in 2006 as being active at a single digit micromolar level against the HCV NS5B RNA-dependent RNA polymerase (RdRp), and 10-fold less potent in the HCV subgenome replication assay in Huh-7 cells (**205**, Figure 16).^174^

Replacement of the C6 benzyl group of *S*-DABOs with a cyclohexylmethyl moiety led to the *S*-DACOs, a series of 4(3*H*)-pyridinone analogues endowed with potent anti-HIV-1 activity in infected cells and against HIV-1 RT (see above, Figure 14).^44^ From 2015, some of these compounds were found to be active against a panel of flaviviruses such as HCV,^175^ dengue virus (DENV),^176^ and Zika virus (ZIKV).^177^ Two *S*-DACOs, **206** and **207**, with nanomolar potency against HIV-1 were shown to inhibit HCV replication in Huh 7.5.1 cells at sub-micromolar level, although with some cytotoxicity (Figure 16).^175^ In the series of the [(5′-aryl-3′-pyrazolyl)methyl]thio *S*-DACOs^129^ (see Figure 14), a proper decoration of the phenylpyrazole scaffold furnished two compounds, **208** and **209**, with reduced anti-HIV-1 potency if compared with the unsubstituted ones and showing inhibition of DENV cytopathogenicity on Vero cells in the range 6.8–17.5 μM for the four serotypes (Figure 16).^176^ Another *S*-DACO (**210**) with the 2-[*N*-(3,5-difluorophenylacetamido)methyl]thio chain at C2, the ethyl group at C5, and the cyclohexyl group at C6, was able to inhibit both HCV infection (by targeting the viral NS5B RdRp) and ZIKV replication at a low micromolar level without apparent cytotoxicity (Figure 16).^177^ It is noteworthy that although active against HCV, the corresponding 6-cyclohexylmethyl counterpart **211** was inactive (up to 50 μM) in the same anti-ZIKV assay. Compound **210** was also able to block the ZIKV-induced plaque formation and to directly inhibit ZIKV RdRp activity.

After the disappointing results obtained with the 2-{[(*N*-phenylcarbamoyl)methyl]thio}-4(3*H*)-pyrimidinone derivatives as HIV-1 replication inhibitors, Liu and co-workers tested these compounds as anti-influenza agents *in vitro*.^178^ Among the synthesized compounds, only **212** (Figure 16) proved to be potent at the single-digit micromolar level in a colorimetric formazan-based MTS assay against influenza A/H1N1 and A/H3N2 strains but showed no inhibitory activity toward the influenza B virus. This compound was less potent than oseltamivir and about equally potent to ribavirin against the H1N1 and H3N2 flu strains but less cytotoxic. Like oseltamivir and ribavirin, **212** was completely inactive against the influenza B strain. These results pave the way for additional possible applications of the whole *S*-DABO class.

More recently, a series of new pyrimidine thioethers, related to *S*-DABO intermediates, have shown antidepressant/anxiolytic, performance enhancing, and nootropic properties based on *in vivo* studies.^179^ Compounds **213** and **214** (Figure 16) increased motor and exploratory search activity and showed a higher interaction frequency and better results in a sucrose preference test in mice affected by social depression, with respect to the untreated mice. Moreover, **213** and **214** showed minimal acute toxicity, lower than that of the positive control fluoxetine.

## 16 Conclusions

**Figure 17:**
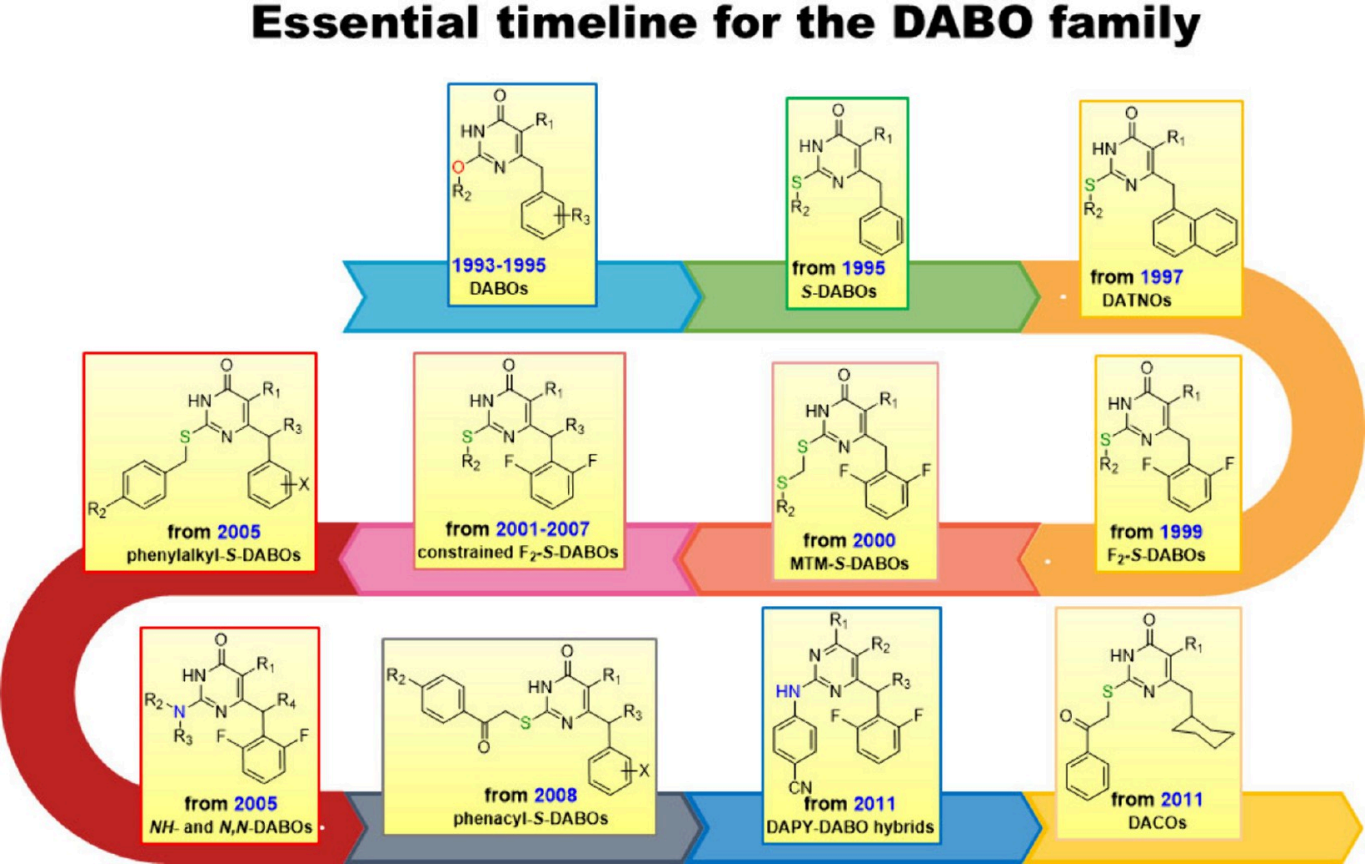
Essential timeline to illustrate the chronological development of DABOs.

During the 30-year DABO history (see the essential timeline in Figure 17), it has emerged that, depending on the substitution inserted at the sulfur/amino atom at C2, the final DABO molecule can resemble either HEPT-like or DABO-like SAR, principally around the best group to be inserted at C5 and the best substitution on the C6 arylalkyl ring. In particular,

- when the sulfur/nitrogen atom at C2 carries a linear, branched, or cyclic aliphatic group or an arylalkyl group, the highest anti-HIV-1 potency is typically obtained with the presence of a methyl group at C5 and a 2,6-difluorobenzyl residue at C6, better when substituted at the α-position with a methyl/methoxy group;
- when the sulfur/nitrogen atom at C2 is linked to a phenacyl or (methylthio)methyl group or congeners, the best substitutions at C5 and C6 seem to be the ethyl/isopropyl at C5 and the 2-chloro-6-fluoro- or 3,5-dimethylbenzyl moiety at C6.

In detail, the 2,6-dihalobenzyl group at C6 linked to the methoxy substituent at the benzylic α-position (as a molecular stiffener) with the *R* configuration turns out to be the decoration of *S*-, *NH*-, and *N*,*N*-DABOs that guarantees maximum potency against WT HIV-1 (at picomolar level) and excellent inhibition of the tested resistant mutants (at nanomolar level). When compared to other clinically approved NNRTIs such as etravirine, rilpivirine, and doravirine^180^ (Figure 1), the best DABO derivatives display similar potency against both WT and mutant HIV-1 strains, including clinical isolates, and acceptable cytotoxicity based on *in vitro* assays but limited water solubility.

**Figure 18:**
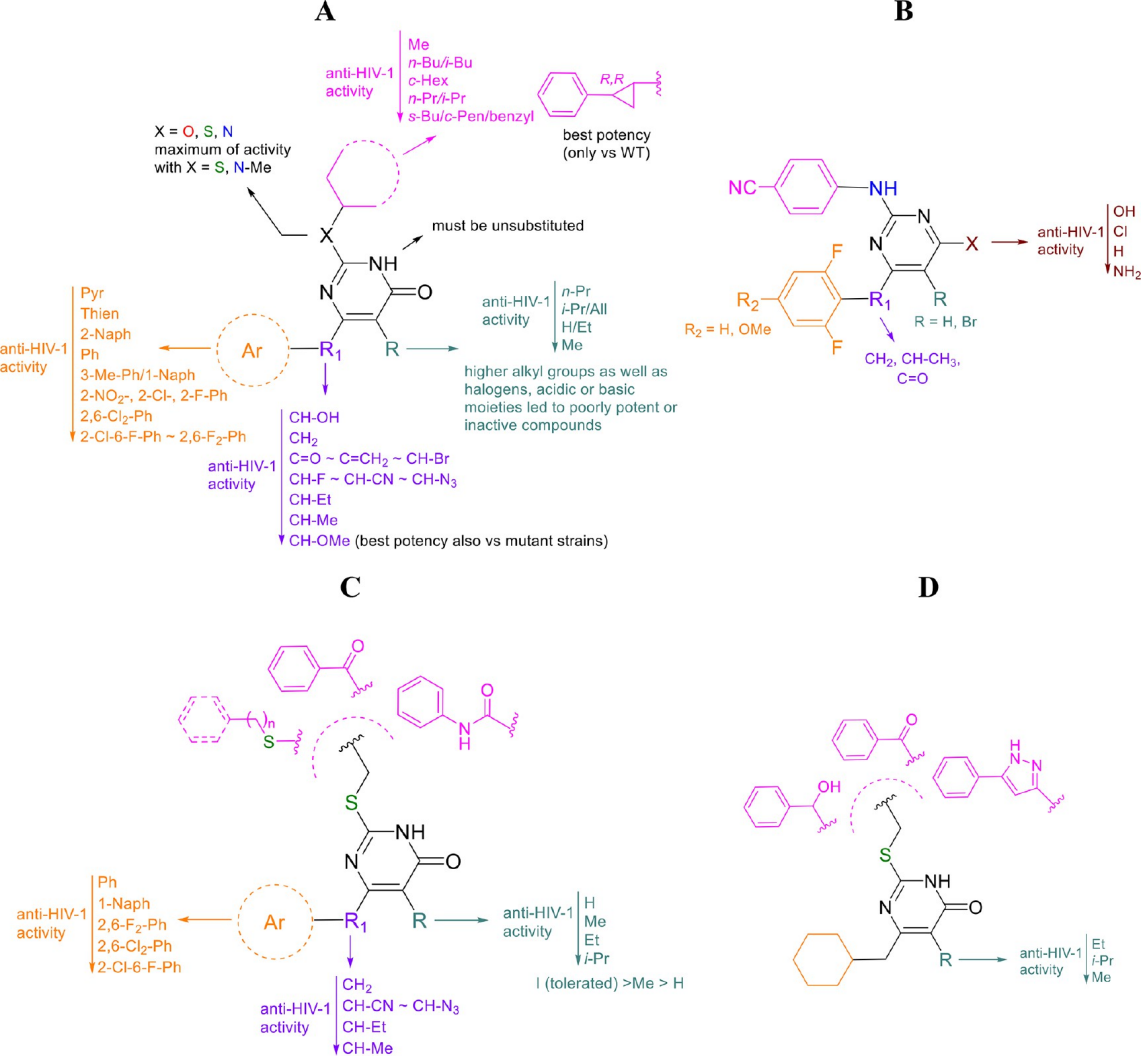
SAR graphical summary of (A) C2-alkoxy-, C2-alkylthio-, C2-arylalkyl-, and C2-amino-DABOs; (B) DAPY/DABO hybrids; (C) C2-aroylalkyl- and C2-(aryl)alkylthiomethyl-*S*-DABOs; and (D) *S*-DACOs.

The SAR graphical summary of four general structures belonging to the DABO family (C2-alkoxy-, C2-alkylthio-, C2-arylalkyl-, and C2-amino-DABOs; DAPY/DABO hybrids; C2-aroylalkyl-, C2-(aryl)alkylthiomethyl-*S*-DABOs; and *S*-DACOs) is depicted in Figure 18.

Moreover, the DABO SAR data lead to some critical issues. First, the problem of effective enantioseparation or enantioselective synthesis of these compounds still remains unsolved along with a cost-effective method for their purification. We successfully reported both methods to obtain pure enantiomers,^35,116,120−123^ but the optimization of this issue is essential for the scalable production of the hit compounds.

Furthermore, ADME weaknesses may also impact the clinical development of these hit compounds. These compounds carry many methyl groups, and this means multiple variants of their hydroxylation by cytochrome P450 isoforms. This drives the potential for rapid inactivation of the compound during first-pass metabolism. From this point of view, *N*,*N*-DABOs seems to be the least sensitive class, because for instance *N*^2^-demethylation of **119** (Figure 11) or **168** (Figure 12) leads to the corresponding *NH*-DABOs without the methyl group on the N atom at C2 but still active as anti-HIV-1 agents. Maybe, C5 and α-benzyl methyl groups may be replaced by some other bioisosteric and about equally lipophilic small groups to increase the metabolic stability of the resulting compounds. For example, the insertion of the methoxy instead of the methyl group at the α-benzylic position increased the potency of the compounds against the HIV-1 resistant mutants, with a lower drop of activity with respect to their effectiveness against WT HIV-1, and at the same time improved their stability toward phase I metabolic inactivation.^116^

In this Perspective, future and further medicinal chemistry optimization efforts on the DABO family should take into account the following:

a. **Increased structural adaptability**. Despite the advantage obtained by applying conformational rigidity to the original DABO structure through the insertion of a small substituent at the α-benzylic position associated with the C5-methyl substitution, a degree of flexibility must be maintained and is essential to counteract HIV mutations, as demonstrated by the replacement of the C6-benzyl with the cyclohexylmethyl group (see the DACO series such as **177**, **180**, and **181** in Figure 14). In general, NNRTIs with a slightly higher number of rotatable bonds show a better activity profile against resistant strains. Structural modifications such as rotatable dihedral angles enhance binding affinity and stability against mutations, providing insight into the design of more adaptable drugs. On the other hand, excessive flexibility in the chemical structure could lead to decreased selectivity of action of compound toward its own specific target.
b. **Solvent–protein interface and binding site innovations.** Modifications to the solvent–protein interface improve drug solubility and ADME profile, while identifying alternative and dual binding sites on RT broadens the potential for effective, mutation-resistant drug design. A very recent successful example of this approach has been reported with DAPYs, in a study in which the flexibility principle has also been applied.^181^
c. **Modification at the C4–C5 position of the pyrimidine ring.** The insertion of a fused heterocycle at the C4–C5 position of the pyrimidine nucleus, as well as the introduction of a heterocyclic ring at the C5 position, are two chemical modifications applied to other NNRTIs to obtain increased potency and/or lower cytotoxicity, which might be introduced in the DABO family.^182,183^
d. **Extended-Release and Preventative Microbicide Applications**. Long-acting NNRTI formulations such as the **116** (Figure 11) vaginal ring are promising for HIV prevention, providing controlled release at transmission sites and improving adherence compared to daily oral doses.^93−95^ This strategy is particularly valuable in settings where oral adherence rates are low, as demonstrated in African clinical trials.

Each approach emphasizes not only combating HIV resistance but also minimizing side effects and improving patient compliance, ultimately enhancing the efficacy and safety of HIV treatment and prevention strategies.

All of these points may be recognized as conclusions, but at the same time, they show the direction for further investigations in this field, targeting an introduction in clinical practice of novel NNRTIs with an extended pharmacokinetic profile.

## Dedication

Dedicated to the memory of Prof. Marino Artico.

## Abbreviations Used

ADAT: 1,3,5-triazine-2(1*H*)-one
*AN*: *Aspergillus niger*
ASFV: African swine fever virus
AZT: azidothymidine/zidovudine
*BC*: *Bacillus cereus*
BHAP: bis-heteroaryl-piperazine
BRAF: v-Raf murine sarcoma viral oncogene homologue B
*BS*: *Bacillus subtilis*
*CA*: *Candida albicans*
CC_50_: cytotoxic concentration of compound able to reduce the viability by 50%
CCID_50_: cell culture infectious dose 50%
CD_50_: cytotoxic dose in 50% of test subjects
CPE: cytopathic effect
COMBINE: comparative binding energy
DABO: 3,4-dihydro-2-alkoxy-6-benzyl-4-oxopyrimidine
DACO: dihydro-aryl/alkylthio-cyclohexylmethyl-oxopyrimidine
DATA: diaryltriazine
DATNO: dihydro-alkylthio-naphthylmethyl-oxopyrimidine
DAPY: diarylpyrimidine
DAVP: 4-dialkylamino-6-vinylpyrimidine
DENV: dengue virus
ddI: 2′,3′-dideoxyinosine
*EC*: *Escherichia coli*
ED_90_: dose effective in 90% of test subjects
endo-RT: endogenous RT
F_2_-*NH*-DABO: 2-alkyl/arylamino-6-(2,6-difluorobenzyl)-4-(3*H*)-pyrimidinone
F_2_-*N*,*N*-DABO: 2-(*N*,*N*-disubstituted) amino-6-(2,6-difluorobenzyl)-4-(3*H*)-pyrimidinone
HEPT: 1-[(2-hydroxyethoxy)methyl]-6-(phenylthio)thymine
IC_90_: 90% inhibitory concentration
ID_50_: 50% inhibiting dose
MLR: multiple linear regression
*ML*: *Micrococcus luteus*
MoI: multiplicity of infection
MTM: (methylthio)methyl
MTS: 3-(4,5-dimethylthiazol-2-yl)-5-(3-carboxymethoxyphenyl)-2-(4-sulfophenyl)-2*H*-tetrazolium assay
NNBP: non-nucleoside reverse transcriptase inhibitor binding pocket
NS5B: nonstructural protein 5B
NVP: nevirapine
*PA*: *Pseudomonas aeruginosa*
PBMC: peripheral blood mononuclear cells
RdRp: RNA-dependent RNA polymerase
rRT: recombinant reverse transcriptase
RT: reverse transcriptase
RT-SHIV: simian immunodeficiency virus containing the reverse transcriptase
RV: rubella virus
*SA*: *Staphylococcus aureus*
Sb-1: Sabin strain
*S*-DABOC: *S*-DABO cytosine analog family
SEVR: silicone elastomer vaginal ring
SI: selectivity index
SIV: simian immunodeficiency virus
SV: Sindbis virus
SVM: support vector machine
TC_50_: median toxic concentration
TD_50_: median toxic dose
UBHA: uracil-based hydroxamic acid
VSV: vesicular stomatitis virus
ZIKV: Zika virus
